# Analysis of global control of *Escherichia coli* carbohydrate uptake

**DOI:** 10.1186/1752-0509-1-42

**Published:** 2007-09-13

**Authors:** Andreas Kremling, Katja Bettenbrock, Ernst Dieter Gilles

**Affiliations:** 1 Max-Planck-Institut Magdeburg, Systems Biology, Sandtorstr. 1, 39106 Magdeburg, Germany

## Abstract

**Background:**

Global control influences the regulation of many individual subsystems by superimposed regulator proteins. A prominent example is the control of carbohydrate uptake systems by the transcription factor Crp in *Escherichia coli*. A detailed understanding of the coordination of the control of individual transporters offers possibilities to explore the potential of microorganisms e.g. in biotechnology.

**Results:**

An o.d.e. based mathematical model is presented that maps a physiological parameter – the specific growth rate – to the sensor of the signal transduction unit, here a component of the bacterial phosphotransferase system (PTS), namely EIIA^*Crr*^. The model describes the relation between the growth rate and the degree of phosphorylation of EIIA ^*crr *^for a number of carbohydrates by a distinctive response curve, that differentiates between PTS transported carbohydrates and non-PTS carbohydrates. With only a small number of kinetic parameters, the model is able to describe a broad range of experimental steady-state and dynamical conditions.

**Conclusion:**

The steady-state characteristic presented shows a relationship between the growth rate and the output of the sensor system PTS. The glycolytic flux that is measured by this sensor is a good indicator to represent the nutritional status of the cell.

## Background

Mathematical models of cellular systems describing metabolism, signal transduction and gene expression are becoming more and more important for the understanding of the underlying molecular processes. Since the earliest work to elucidate the molecular nature of regulatory structures by J. Monod, the knowledge of the detailed interactions between the components that are responsible for carbohydrate uptake in *Escherichia coli *is steadily increasing. Although current research on individual uptake systems like glucose still reveals new players that maybe play a role in local control [1], the knowledge of individual uptake systems is rich and is used as a basis to set up mathematical models to describe and analyze the properties of the control circuits. E.g. for the lactose uptake system in *E. coli*, it was shown that the autocatalytic action of inducer allolactose is responsible for the existence of multi-stationarity [2]. Such nonlinear properties of sub-networks are often described and assigned to a certain functionality of the system. The understanding of how different stimuli of the same type – in this study carbohydrates – are sensed by the cells and how these different signals are processed is still lacking. Here, we used experimental data published by our group [3,4] to elucidate and characterize such a global control circuit, that is, a regulatory scheme, that senses a physiological parameter like the specific growth rate and maps it to the degree of phosphorylation of the intracellular component EIIA^*Crr*^. EIIA^*Crr *^is a component of the phosphoenolpyruvate (PEP): carbohydrate phosphotransferase system (PTS). The PTS is not only a transport system for a number of carbohydrates but also acts as a sensory system. Sensor elements like the PTS can be seen as logic elements that process external stimuli into intracellular signals. High fluxes through the glycolysis, corresponding to high growth rates result in a low degree of phosphorylation of EIIA^*Crr*^. At first view, this is surprising, since, assuming a linear reaction chain, high fluxes result in high pool concentrations, based on the (normally) monotone dependency of the reaction rate on the substrate concentration. The PTS together with the glycolysis can now be seen as an element that allows a transformation of high fluxes into a low pool concentration. This is not only due to the existence of two complementary pools like EIIA^*Crr *^and its phosphorylated form, but as we will show, depends strongly on the flux distribution at the PEP node. High fluxes through the glycolysis result in low values of the phosphorylated form of EIIA^*Crr *^while low fluxes indicate a hunger situation and the global transcription factor cAMP·Crp is activated.

Interestingly, the relationship between growth rate and degree of phosphorylation of EIIA^*Crr *^could be seen in various growth situations of the wild type strain growing on single substrates like glucose, lactose, and glycerol and for growth on mixtures of substrates, and of a PtsG deletion mutant strain missing *ptsG*, a gene that is central for glucose transport.

### Carbohydrate uptake by E. coli

The PTS of *E. coli *consist of two common cytoplasmatic proteins, EI (enzymeI) and HPr (histidine containing protein), as well as of an array of carbohydrate-specific EII (enzymeII) complexes. E.g. for glucose uptake, a phosphoryl group is transferred from phosphoenolpyruvate (PEP) through EI, HPr, EIIA^*Crr*^, PtsG (also known as EIICB^*Glc*^, that is the membranstanding transport protein) and finally to the substrate. Since all components of the PTS, depending on their phosphorylation status, can interact with various key regulator proteins the output of the PTS is represented by the degree of phosphorylation of the proteins involved in phosphoryl group transfer.

**Figure 1:**
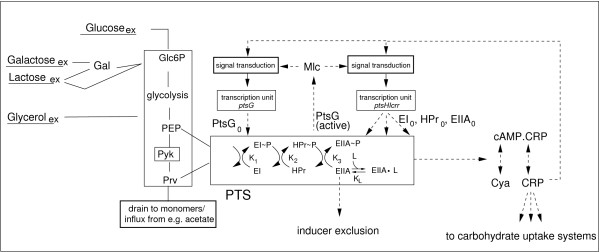
A rough scheme of the interactions of the PTS. The degree of phosphorylation of the PTS proteins is influenced by the flux through glycolysis and the overall concentration of the proteins. The respective genes are subject to transcriptional control by several transcriptions factors, e.g. Mlc and Crp and post-transcriptional control (not shown). The degree of phosphorylation of EIIA^*Crr *^is furthermore influenced by interactions with other proteins (L) during inducer exclusion. In case of a PTS sugar, the phosphoryl group from EIIA^*Crr *^is transferred to the transported sugar. E.g. glucose appears as glucose 6-phosphate inside the cell.

Figure 1 gives a rough sketch on the components that influence the degree of phosphorylation of protein EIIA^*Crr*^: (i) Metabolic fluxes through the glycolysis. Extracellular glucose is taken up by PtsG and enters into the cell as glucose 6-phosphate. Other carbohydrates enter glycolysis at the same node (e.g. galactose and lactose) or at other nodes (e. g. glycerol at triose phosphate). The carbohydrates are further metabolized by glycolytic reactions. At node PEP, the flux is subdivided. One part is converted to pyruvate by pyruvate kinase while the remainder part is converted to pyruvate by the PTS. Other fluxes from or to PEP or pyruvate are marginal and hence are not considered in this study. Fluxes from e.g. acetate uptake enter gluconeogenesis via pyruvate or from TCA. (ii) Overall concentration of the PTS proteins. The expression of the *pts *genes (*ptsHIcrr, ptsG*) is subject to control by various regulators, with Mlc and Crp being the most important ones. Mlc is a repressor that is active if no glucose is present in the medium. A possible mechanism of the interaction between Mlc and EIICB^*Crr *^is described in [1]. Crp is a global regulator that is involved in the regulation of a number of genes; it is activated by cAMP. cAMP is synthesized from ATP by adenylate cyclase (Cya). Both proteins, Crp and Cya, are subject to control by the cAMP·Crp complex itself. Recent investigations indicate that the *ptsG *transcript is subject to post-transcriptional control by a small RNA (sRNA) regulator SgrS which is induced at different stress conditions, e.g. glucose-phosphate stress. This stress occurs when cells accumulate glucose 6-phosphate or the glucose analog a-methyl-glucoside 6-phosphate and leads to the degradation of PtsG mRNA [5-7]. (iii) Another parameter that determines the degree of phosphorylation of protein EIIA^*Crr *^is the overall equilibrium constant *K*_*pts *_that links the PEP/pyruvate ratio to the degree of phosphorylation. Figure 1 considers a general case where the phosphoryl group is transferred from PEP to EIIA^*Crr*^. Furthermore, EIIA^*Crr *^is considered to exist in a free form and in a form bound to a protein L involved in carbohydrate transport or metabolism (lactose permease, glycerol kinase). Then, the equilibrium constant *K*_*pts *_can be determined as:

Kpts=K1⋅K2⋅K3⋅(L+KL)KL
 MathType@MTEF@5@5@+=feaafiart1ev1aaatCvAUfKttLearuWrP9MDH5MBPbIqV92AaeXatLxBI9gBaebbnrfifHhDYfgasaacH8akY=wiFfYdH8Gipec8Eeeu0xXdbba9frFj0=OqFfea0dXdd9vqai=hGuQ8kuc9pgc9s8qqaq=dirpe0xb9q8qiLsFr0=vr0=vr0dc8meaabaqaciaacaGaaeqabaqabeGadaaakeaacqWGlbWsdaWgaaWcbaGaemiCaaNaemiDaqNaem4Camhabeaakiabg2da9maalaaabaGaem4saS0aaSbaaSqaaiabigdaXaqabaGccqGHflY1cqWGlbWsdaWgaaWcbaGaeGOmaidabeaakiabgwSixlabdUealnaaBaaaleaacqaIZaWmaeqaaOGaeyyXICTaeiikaGIaemitaWKaey4kaSIaem4saS0aaSbaaSqaaiabdYeambqabaGccqGGPaqkaeaacqWGlbWsdaWgaaWcbaGaemitaWeabeaaaaaaaa@49AA@

with *K*_1_, *K*_2_, *K*_3_, *K*_*L *_being the respective equilibrium constants from the single reactions shown in Figure 1. If EIIA^*Crr *^is bound to lactose permease or glycerol kinase, it acts as an inhibitor that prevents uptake and/or metabolism of the substrate, an effect that is called inducer exclusion.

The intention of this contribution is to develop a model with a small number of state variables and parameters to work out the basic principles for the understanding of the sensor function. Nearly all parameters could be determined from experiments (for material and methods, [see Additional file 1]). The core of the model describes the mapping of the specific growth characteristics represented by the carbohydrate uptake rates to the degree of phosphorylation of the PTS component EIIA^*Crr*^. The kinetic properties of the sensor which at the same time is a transport system are characterized and the output of the sensor is mapped to the rate of synthesis of genes that are under control of transcription factor cAMP·Crp. In this way, a closed loop is established that precisely adjusts the respective transport protein to maintain the incoming flux. The results are used to predict the transient behavior during glucose/glucose 6-phosphate diauxic growth and glucose/lactose diauxic growth. Finally, we also show that the approach can be generalized for other main growth substrates like acetate. In the end, a comparison with a corresponding detailed model on catabolite repression [3] is performed.

## Results and discussion

### Sensor characteristics

**Figure 2:**
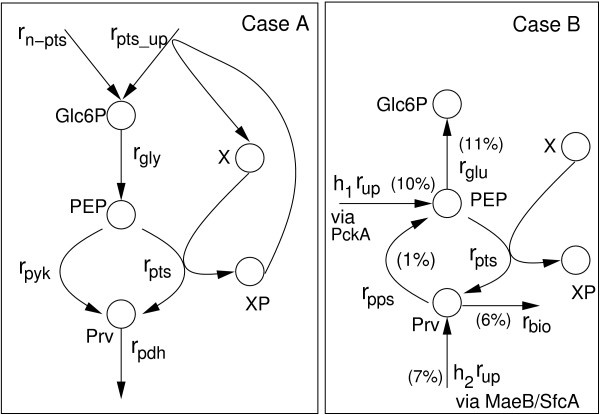
Reactions schemes that describe the fluxes through glycolysis and the PEP/pyruvate node. Left: *Case A*. Growth on glycolytic PTS substrate and non-PTS substrates. State variable *X *represents all PTS components. Right: *Case B*. Growth on gluconeogenetic substrates. Values in parenthesis indicate the flux distribution during growth on acetate [10] in % of the acetate uptake. Main routes to PEP and pyruvate are via PckA (PEP carboxykinase) and MaeB/SfcA (malate dehydrogenase).

First, the steady-state properties of the core system, comprising glycolytic and PTS reactions, are analyzed. Predictions with the model are performed and compared with experimental data. Based on the molecular details, two situations are considered (Figure 2). *Case A *considers growth on glycolytic substrates, that is, carbohydrates that feed into glycolysis. This includes growth on PTS and on non-PTS substrates. E.g. glucose enters the cell by a PTS as glucose 6-phosphate, while lactose is a non-PTS substrate. Intracellular lactose is split into glucose and galactose by LacZ. The resulting intracellular glucose is phosphorylated by PtsG and/or by glukokinase. Galactose, too, is further metabolized and both enter via glucose 6-phosphate into glycolysis. In case of lactose, EIIA^*Crr*^mediates inducer exclusion by binding to lactose permease. This alters the overall equilibrium constant as described above.

The scheme simplifies the biological knowledge on metabolism and gene expression by lumping together reactions and components. In case A, carbohydrate uptake is represented by reactions *r*_*pts_up *_for a PTS carbohydrate and *r*_*n*-*pts *_for a non-PTS carbohydrate. Glycolysis is simply represented by metabolite *Glc*6*P*. The flux at node *PEP *is subdivided into *r*_*pyk *_for pyruvate kinase and *r*_*pts*_. The drain from pyruvate (*Prv*) to other parts of the central metabolism is represented by *r*_*pdh*_. Since other fluxes from or to PEP and pyruvate are rather marginal they are not considered in the model. Proteins EI, HPr, and EIIA^*Crr *^of the PTS are represented by only one component *X *that exists either in the unphosphorylated form *X *or in the phosphorylated form *XP*. In case of a PTS carbohydrate, *XP *is used for transport via *r*_*pts_up*_.

*Case B *considers gluconeogenetic substrates which feed into TCA or into other central metabolites below the PEP/pyruvate branch. Here, PEP and pyruvate are produced by a number of different reactions, e.g. from the TCA or via Acetyl CoA. Among these, PEP synthase (Pps) is active converting pyruvate directly to PEP. For substrates that enter TCA, two pathways are known that connect TCA and glycolysis: PckA (PEP carboxykinase) connects oxaloacetate and PEP, MaeB/SfcA (malate dehydrogenase) connect malate and pyruvate. These fluxes are represented by *h*_1 _*r*_*up *_and *h*_2 _*r*_*up*_, respectively, with *h*_1 _and *h*_2 _are numbers between zero and one, representing a fraction of the uptake rate *r*_*up*_. In a number of subsequent gluconeogenetic reaction steps (*r*_*glu*_), PEP is then converted to glucose 6-phosphate.

Based on the knowledge presented so far, a simplified model structure is suggested that is able to simulate the different cases proposed above.

#### Glycolytic substrates

As was shown in a previous study [8], the metabolic part of the considered network reaches the steady-state very fast. Therefore, the steady-state assumption will be used as a starting point for model analysis. For *G*6*P*, *PEP*, *Prv *and the protein that represents the PTS, *XP*, the following equations that describe the dynamics are obtained from the scheme:

G˙6P=rn−pts+rpts_up−rgly
 MathType@MTEF@5@5@+=feaafiart1ev1aaatCvAUfKttLearuWrP9MDH5MBPbIqV92AaeXatLxBI9gBaebbnrfifHhDYfgasaacH8akY=wiFfYdH8Gipec8Eeeu0xXdbba9frFj0=OqFfea0dXdd9vqai=hGuQ8kuc9pgc9s8qqaq=dirpe0xb9q8qiLsFr0=vr0=vr0dc8meaabaqaciaacaGaaeqabaqabeGadaaakeaacuWGhbWrgaGaaiabiAda2iabdcfaqjabg2da9iabdkhaYnaaBaaaleaacqWGUbGBcqGHsislcqWGWbaCcqWG0baDcqWGZbWCaeqaaOGaey4kaSIaemOCai3aaSbaaSqaaiabdchaWjabdsha0jabdohaZjabc+faFjabdwha1jabdchaWbqabaGccqGHsislcqWGYbGCdaWgaaWcbaGaem4zaCMaemiBaWMaemyEaKhabeaaaaa@4ADC@

P˙EP=2rgly−rpyk−rpts
 MathType@MTEF@5@5@+=feaafiart1ev1aaatCvAUfKttLearuWrP9MDH5MBPbIqV92AaeXatLxBI9gBaebbnrfifHhDYfgasaacH8akY=wiFfYdH8Gipec8Eeeu0xXdbba9frFj0=OqFfea0dXdd9vqai=hGuQ8kuc9pgc9s8qqaq=dirpe0xb9q8qiLsFr0=vr0=vr0dc8meaabaqaciaacaGaaeqabaqabeGadaaakeaacuWGqbaugaGaaiabdweafjabdcfaqjabg2da9iabikdaYiabdkhaYnaaBaaaleaacqWGNbWzcqWGSbaBcqWG5bqEaeqaaOGaeyOeI0IaemOCai3aaSbaaSqaaiabdchaWjabdMha5jabdUgaRbqabaGccqGHsislcqWGYbGCdaWgaaWcbaGaemiCaaNaemiDaqNaem4Camhabeaaaaa@458A@

P˙rv=rpts+rpyk−rpdh
 MathType@MTEF@5@5@+=feaafiart1ev1aaatCvAUfKttLearuWrP9MDH5MBPbIqV92AaeXatLxBI9gBaebbnrfifHhDYfgasaacH8akY=wiFfYdH8Gipec8Eeeu0xXdbba9frFj0=OqFfea0dXdd9vqai=hGuQ8kuc9pgc9s8qqaq=dirpe0xb9q8qiLsFr0=vr0=vr0dc8meaabaqaciaacaGaaeqabaqabeGadaaakeaacuWGqbaugaGaaiabdkhaYjabdAha2jabg2da9iabdkhaYnaaBaaaleaacqWGWbaCcqWG0baDcqWGZbWCaeqaaOGaey4kaSIaemOCai3aaSbaaSqaaiabdchaWjabdMha5jabdUgaRbqabaGccqGHsislcqWGYbGCdaWgaaWcbaGaemiCaaNaemizaqMaemiAaGgabeaaaaa@4513@

X˙P=rpts−rpts_up
 MathType@MTEF@5@5@+=feaafiart1ev1aaatCvAUfKttLearuWrP9MDH5MBPbIqV92AaeXatLxBI9gBaebbnrfifHhDYfgasaacH8akY=wiFfYdH8Gipec8Eeeu0xXdbba9frFj0=OqFfea0dXdd9vqai=hGuQ8kuc9pgc9s8qqaq=dirpe0xb9q8qiLsFr0=vr0=vr0dc8meaabaqaciaacaGaaeqabaqabeGadaaakeaacuWGybawgaGaaiabdcfaqjabg2da9iabdkhaYnaaBaaaleaacqWGWbaCcqWG0baDcqWGZbWCaeqaaOGaeyOeI0IaemOCai3aaSbaaSqaaiabdchaWjabdsha0jabdohaZjabc+faFjabdwha1jabdchaWbqabaaaaa@40FA@

where *r*_*n*-*pts *_and *r*_*pts_up *_are the systems inputs and are related by the yield coefficients to the specific growth rate. *XP *is the system output. The following conditions will hold for the defined rates in steady-state:

*r*_*pts *_= *r*_*pts_up*_

*r*_*gly *_= *r*_*n*-*pts *_+ *r*_*pts_up*_

*r*_*pdh *_= 2 (*r*_*n*-*pts *_+ *r*_*pts_up*_)

*r*_*pyk *_= 2 *r*_*n*-*pts *_+ *r*_*pts_up*_

The kinetics for the rate laws are kept as simple as possible to describe the experimental data. The rate laws are assumed as follows:

*r*_*gly *_= *k*_*gly *_*G*6*P*

*r*_*pdh *_= *k*_*pdh *_*Prv*

*r*_*pts *_= *k*_*pts*_(*PEP*(*X*_0 _- *XP*) - *K*_*pts *_*Prv XP*)

*r*_*pyk *_= *k*_*pyk *_*PEP f *(*PEP*, ...),

with *X*_0 _is the overall concentration of the PTS protein. The focus of the analysis will be on the branch point at PEP. To elucidate the correct choice of the kinetic rate law for the pyruvate kinase reaction, function *f *is introduced that represents different model variants. Function *f *depends on *PEP *but may also depend on different metabolites in the network.

The steady-state concentrations can be derived from the equations above:

G6P=rn−pts+rpts_upkgly
 MathType@MTEF@5@5@+=feaafiart1ev1aaatCvAUfKttLearuWrP9MDH5MBPbIqV92AaeXatLxBI9gBaebbnrfifHhDYfgasaacH8akY=wiFfYdH8Gipec8Eeeu0xXdbba9frFj0=OqFfea0dXdd9vqai=hGuQ8kuc9pgc9s8qqaq=dirpe0xb9q8qiLsFr0=vr0=vr0dc8meaabaqaciaacaGaaeqabaqabeGadaaakeaacqWGhbWrcqaI2aGncqWGqbaucqGH9aqpdaWcaaqaaiabdkhaYnaaBaaaleaacqWGUbGBcqGHsislcqWGWbaCcqWG0baDcqWGZbWCaeqaaOGaey4kaSIaemOCai3aaSbaaSqaaiabdchaWjabdsha0jabdohaZjabc+faFjabdwha1jabdchaWbqabaaakeaacqWGRbWAdaWgaaWcbaGaem4zaCMaemiBaWMaemyEaKhabeaaaaaaaa@49E8@

Prv=2rn−pts+rpts_upkpdh
 MathType@MTEF@5@5@+=feaafiart1ev1aaatCvAUfKttLearuWrP9MDH5MBPbIqV92AaeXatLxBI9gBaebbnrfifHhDYfgasaacH8akY=wiFfYdH8Gipec8Eeeu0xXdbba9frFj0=OqFfea0dXdd9vqai=hGuQ8kuc9pgc9s8qqaq=dirpe0xb9q8qiLsFr0=vr0=vr0dc8meaabaqaciaacaGaaeqabaqabeGadaaakeaaieGacqWFqbaucqWFYbGCcqWG2bGDcqGH9aqpcqaIYaGmdaWcaaqaaiabdkhaYnaaBaaaleaacqWGUbGBcqGHsislcqWGWbaCcqWG0baDcqWGZbWCaeqaaOGaey4kaSIaemOCai3aaSbaaSqaaiabdchaWjabdsha0jabdohaZjabc+faFjabdwha1jabdchaWbqabaaakeaacqWGRbWAdaWgaaWcbaGaemiCaaNaemizaqMaemiAaGgabeaaaaaaaa@4B8E@

PEP=2rn−pts+rpts_upkpykf
 MathType@MTEF@5@5@+=feaafiart1ev1aaatCvAUfKttLearuWrP9MDH5MBPbIqV92AaeXatLxBI9gBaebbnrfifHhDYfgasaacH8akY=wiFfYdH8Gipec8Eeeu0xXdbba9frFj0=OqFfea0dXdd9vqai=hGuQ8kuc9pgc9s8qqaq=dirpe0xb9q8qiLsFr0=vr0=vr0dc8meaabaqaciaacaGaaeqabaqabeGadaaakeaacqWGqbaucqWGfbqrcqWGqbaucqGH9aqpdaWcaaqaaiabikdaYiabdkhaYnaaBaaaleaacqWGUbGBcqGHsislcqWGWbaCcqWG0baDcqWGZbWCaeqaaOGaey4kaSIaemOCai3aaSbaaSqaaiabdchaWjabdsha0jabdohaZjabc+faFjabdwha1jabdchaWbqabaaakeaacqWGRbWAdaWgaaWcbaGaemiCaaNaemyEaKNaem4AaSgabeaakiabdAgaMbaaaaa@4C74@

XP=kpdhX0−rpts_upkptsPEPkpdh+2Kptskpykfrn−pts+rpts_up2rn−pts+rpts_up
 MathType@MTEF@5@5@+=feaafiart1ev1aaatCvAUfKttLearuWrP9MDH5MBPbIqV92AaeXatLxBI9gBaebbnrfifHhDYfgasaacH8akY=wiFfYdH8Gipec8Eeeu0xXdbba9frFj0=OqFfea0dXdd9vqai=hGuQ8kuc9pgc9s8qqaq=dirpe0xb9q8qiLsFr0=vr0=vr0dc8meaabaqaciaacaGaaeqabaqabeGadaaakeaacqWGybawcqWGqbaucqGH9aqpcqWGRbWAdaWgaaWcbaGaemiCaaNaemizaqMaemiAaGgabeaakmaalaaabaGaemiwaG1aaSbaaSqaaiabicdaWaqabaGccqGHsisldaWcaaqaaiabdkhaYnaaBaaaleaacqWGWbaCcqWG0baDcqWGZbWCcqGGFbWxcqWG1bqDcqWGWbaCaeqaaaGcbaGaem4AaS2aaSbaaSqaaiabdchaWjabdsha0jabdohaZbqabaGccqWGqbaucqWGfbqrcqWGqbauaaaabaGaem4AaS2aaSbaaSqaaiabdchaWjabdsgaKjabdIgaObqabaGccqGHRaWkcqaIYaGmcqWGlbWsdaWgaaWcbaGaemiCaaNaemiDaqNaem4CamhabeaakiabdUgaRnaaBaaaleaacqWGWbaCcqWG5bqEcqWGRbWAaeqaaOGaemOzay2aaSaaaeaacqWGYbGCdaWgaaWcbaGaemOBa4MaeyOeI0IaemiCaaNaemiDaqNaem4CamhabeaakiabgUcaRiabdkhaYnaaBaaaleaacqWGWbaCcqWG0baDcqWGZbWCcqGGFbWxcqWG1bqDcqWGWbaCaeqaaaGcbaGaeGOmaiJaemOCai3aaSbaaSqaaiabd6gaUjabgkHiTiabdchaWjabdsha0jabdohaZbqabaGccqGHRaWkcqWGYbGCdaWgaaWcbaGaemiCaaNaemiDaqNaem4CamNaei4xa8LaemyDauNaemiCaahabeaaaaaaaaaa@8812@

The steady-state equation for *PEP *is given in implicit form since it depends on function *f*. In the following, growth situations on non-PTS and PTS sugars are considered separately.

Equation (17) for the non-PTS case reads

XP=kpdhX0kpdh+Kptskpykf
 MathType@MTEF@5@5@+=feaafiart1ev1aaatCvAUfKttLearuWrP9MDH5MBPbIqV92AaeXatLxBI9gBamXvP5wqSXMqHnxAJn0BKvguHDwzZbqegyvzYrwyUfgarqqtubsr4rNCHbGeaGqiA8vkIkVAFgIELiFeLkFeLk=iY=Hhbbf9v8qqaqFr0xc9pk0xbba9q8WqFfeaY=biLkVcLq=JHqVepeea0=as0db9vqpepesP0xe9Fve9Fve9GapdbaqaaeGacaGaaiaabeqaamqadiabaaGcbaGaemiwaGLaemiuaaLaeyypa0Jaem4AaS2aaSbaaSqaaiabdchaWjabdsgaKjabdIgaObqabaGcdaWcaaqaaiabdIfaynaaBaaaleaacqaIWaamaeqaaaGcbaGaem4AaS2aaSbaaSqaaiabdchaWjabdsgaKjabdIgaObqabaGccqGHRaWkcqWGlbWsdaWgaaWcbaGaemiCaaNaemiDaqNaem4CamhabeaakiabdUgaRnaaBaaaleaacqWGWbaCcqWG5bqEcqWGRbWAaeqaaOGaemOzaygaaaaa@5BBD@

As can be seen cleary, the choice of *f *has a strong influence on the steady-state characteristics: Assuming *f *= 1, that is, the pyruvate kinase reaction is modeled as a first order reaction, *XP *is constant and independent from the uptake rate. This could not be observed in the experiments (see below). Assuming a Michaelis-Menten kinetics, that is, f=1K+PEP
 MathType@MTEF@5@5@+=feaafiart1ev1aaatCvAUfKttLearuWrP9MDH5MBPbIqV92AaeXatLxBI9gBaebbnrfifHhDYfgasaacH8akY=wiFfYdH8Gipec8Eeeu0xXdbba9frFj0=OqFfea0dXdd9vqai=hGuQ8kuc9pgc9s8qqaq=dirpe0xb9q8qiLsFr0=vr0=vr0dc8meaabaqaciaacaGaaeqabaqabeGadaaakeaacqWGMbGzcqGH9aqpdaWcaaqaaiabigdaXaqaaiabdUealjabgUcaRiabdcfaqjabdweafjabdcfaqbaaaaa@356D@, the steady-state concentration of *PEP *can be calculated via Equation (16):

PEP=2rn−ptskpykf=2rn−ptskpyk1K+PEP
 MathType@MTEF@5@5@+=feaafiart1ev1aaatCvAUfKttLearuWrP9MDH5MBPbIqV92AaeXatLxBI9gBaebbnrfifHhDYfgasaacH8akY=wiFfYdH8Gipec8Eeeu0xXdbba9frFj0=OqFfea0dXdd9vqai=hGuQ8kuc9pgc9s8qqaq=dirpe0xb9q8qiLsFr0=vr0=vr0dc8meaabaqaciaacaGaaeqabaqabeGadaaakeaacqWGqbaucqWGfbqrcqWGqbaucqGH9aqpdaWcaaqaaiabikdaYiabdkhaYnaaBaaaleaacqWGUbGBcqGHsislcqWGWbaCcqWG0baDcqWGZbWCaeqaaaGcbaGaem4AaS2aaSbaaSqaaiabdchaWjabdMha5jabdUgaRbqabaGccqWGMbGzaaGaeyypa0ZaaSaaaeaacqaIYaGmcqWGYbGCdaWgaaWcbaGaemOBa4MaeyOeI0IaemiCaaNaemiDaqNaem4CamhabeaaaOqaaiabdUgaRnaaBaaaleaacqWGWbaCcqWG5bqEcqWGRbWAaeqaaOWaaSaaaeaacqaIXaqmaeaacqWGlbWscqGHRaWkcqWGqbaucqWGfbqrcqWGqbauaaaaaaaa@5808@

⇒PEP=2Krn−ptskpyk−2rn−pts
 MathType@MTEF@5@5@+=feaafiart1ev1aaatCvAUfKttLearuWrP9MDH5MBPbIqV92AaeXatLxBI9gBaebbnrfifHhDYfgasaacH8akY=wiFfYdH8Gipec8Eeeu0xXdbba9frFj0=OqFfea0dXdd9vqai=hGuQ8kuc9pgc9s8qqaq=dirpe0xb9q8qiLsFr0=vr0=vr0dc8meaabaqaciaacaGaaeqabaqabeGadaaakeaafaqabeqacaaabaGaeyO0H4nabaGaemiuaaLaemyrauKaemiuaaLaeyypa0ZaaSaaaeaacqaIYaGmcqWGlbWscqWGYbGCdaWgaaWcbaGaemOBa4MaeyOeI0IaemiCaaNaemiDaqNaem4CamhabeaaaOqaaiabdUgaRnaaBaaaleaacqWGWbaCcqWG5bqEcqWGRbWAaeqaaOGaeyOeI0IaeGOmaiJaemOCai3aaSbaaSqaaiabd6gaUjabgkHiTiabdchaWjabdsha0jabdohaZbqabaaaaaaaaaa@4DCB@

Since *k*_*pyk*_, in this case, is the maximal reaction rate of *r*_*pyk*_, *PEP *is an increasing monotone function in dependency on the uptake rate *r*_*n*-*pts*_. Interestingly, this leads to values for *XP *that increase for increasing uptake rates. This result is again contradictory to the observed experimental results.

Equation (17) for PTS substrates reads:

XP=kpdhX0−rpts_upkptsPEPkpdh+2Kptskpykf
 MathType@MTEF@5@5@+=feaafiart1ev1aaatCvAUfKttLearuWrP9MDH5MBPbIqV92AaeXatLxBI9gBaebbnrfifHhDYfgasaacH8akY=wiFfYdH8Gipec8Eeeu0xXdbba9frFj0=OqFfea0dXdd9vqai=hGuQ8kuc9pgc9s8qqaq=dirpe0xb9q8qiLsFr0=vr0=vr0dc8meaabaqaciaacaGaaeqabaqabeGadaaakeaacqWGybawcqWGqbaucqGH9aqpcqWGRbWAdaWgaaWcbaGaemiCaaNaemizaqMaemiAaGgabeaakmaalaaabaGaemiwaG1aaSbaaSqaaiabicdaWaqabaGccqGHsisldaWcaaqaaiabdkhaYnaaBaaaleaacqWGWbaCcqWG0baDcqWGZbWCcqGGFbWxcqWG1bqDcqWGWbaCaeqaaaGcbaGaem4AaS2aaSbaaSqaaiabdchaWjabdsha0jabdohaZbqabaGccqWGqbaucqWGfbqrcqWGqbauaaaabaGaem4AaS2aaSbaaSqaaiabdchaWjabdsgaKjabdIgaObqabaGccqGHRaWkcqaIYaGmcqWGlbWsdaWgaaWcbaGaemiCaaNaemiDaqNaem4CamhabeaakiabdUgaRnaaBaaaleaacqWGWbaCcqWG5bqEcqWGRbWAaeqaaOGaemOzaygaaaaa@60BE@

Differences for PTS and non-PTS substrates can be seen in the numerator that is always smaller in case of growth on PTS substrates. Since the denominator is always larger than in the case of non-PTS substrates, the curve of the PTS substrates will always be below the curves for non-PTS substrates.

To describe the available experimental data for growth on PTS and non-PTS substrates (Table 2 in [Additional file 1]), parameters were estimated by a least square approach. A reasonable fit could be obtained with

*f *= *f*_1_(*G*6*P*)·*f*_2_(*PEP*) = *G*6*P*^*n*^·*PEP*^*m*^.

Since the pyruvate kinase in *E. coli *is a tetramer that needs activation from a glycolytic metabolite (in *E. coli *PykF is strongly activated by fructose 1,6-bis-phosphate, that is not included in the model, but is represented by glucose 6-phosphate instead), values for *n *> 1, *m *≥ 1 are analyzed. Equation (1) relates the overall PTS constant *K*_*pts *_to individual reactions steps. Since measurements of proteins that influence *K*_*pts *_are not available, *K*_*pts *_represents a mean value for different situations considered in the experiments. For parameter identification 31 data points are considered, values *n *= 2, *m *= 1 are fixed and values for *K*_*pts *_and *X*_0 _are taken from literature (Table 6 in the [Additional file 1]); so, four parameters are estimated: *k*_*gly*_, *k*_*pyk*_, *k*_*pts*_, and *k*_*pdh*_.

The standard deviation σ^
 MathType@MTEF@5@5@+=feaafiart1ev1aaatCvAUfKttLearuWrP9MDH5MBPbIqV92AaeXatLxBI9gBaebbnrfifHhDYfgasaacH8akY=wiFfYdH8Gipec8Eeeu0xXdbba9frFj0=OqFfea0dXdd9vqai=hGuQ8kuc9pgc9s8qqaq=dirpe0xb9q8qiLsFr0=vr0=vr0dc8meaabaqaciaacaGaaeqabaqabeGadaaakeaaiiGacuWFdpWCgaqcaaaa@2E86@ of the measured data for the degree of phosphorylation of EIIA^*Crr *^is estimated with the degree of freedom *df *= 31 (data points) -4 (parameters):

σ^=∑(XPi−XPmi)2df=0.013.
 MathType@MTEF@5@5@+=feaafiart1ev1aaatCvAUfKttLearuWrP9MDH5MBPbIqV92AaeXatLxBI9gBaebbnrfifHhDYfgasaacH8akY=wiFfYdH8Gipec8Eeeu0xXdbba9frFj0=OqFfea0dXdd9vqai=hGuQ8kuc9pgc9s8qqaq=dirpe0xb9q8qiLsFr0=vr0=vr0dc8meaabaqaciaacaGaaeqabaqabeGadaaakeaaiiGacuWFdpWCgaqcaiabg2da9maakaaabaWaaSaaaeaadaaeabqaaiabcIcaOiabdIfayjabdcfaqnaaBaaaleaacqWGPbqAaeqaaOGaeyOeI0IaemiwaGLaemiuaa1aaSbaaSqaaiabd2gaTnaaBaaameaacqWGPbqAaeqaaaWcbeaakiabcMcaPmaaCaaaleqabaGaeGOmaidaaaqabeqaniabggHiLdaakeaacqWGKbazcqWGMbGzaaaaleqaaOGaeyypa0JaeGimaaJaeiOla4IaeGimaaJaeGymaeJaeG4mamJaeiOla4caaa@4847@

**Figure 3:**
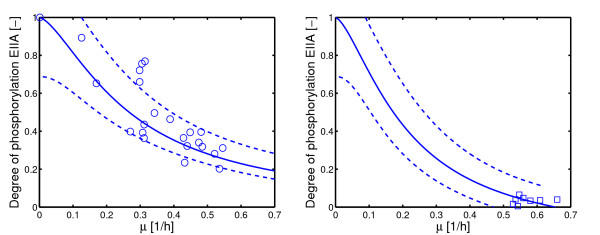
Course of the degree of phosphorylation of *X *in dependence on the growth rate. Since most experiments are performed with lactose and glucose, the specific growth rate can be converted with (nearly) the same yield coefficient (on a molar basis) into an uptake rate. Left: Growth on non-PTS carbohydrates (lactose and glycerol, wild type and mutant strain BKG47); Right: Growth on PTS carbohydrates (glucose, wild type). Dashed lines indicate a 95% confidence interval based on the simulated and the experimental data. The calculation is based on a linearization around the estimated parameters, therefore, it is not exptected that all the data can be found in-between the two limits.

Based on the maximal value *X*_0 _this corresponds to 13%. Figure 3 shows the results of the parameter estimation. Parameter values and confidence regions are summarized in Table 6 in [Additional file 1]. Dashed lines mark a 95% confidence band of the simulation based on the linearized system (linearized with respect to the parameters; for details [Additional file 1]).

A robustness analysis was performed as described earlier [8]. Instead of presenting individual sensitivities, a ranking of all sensitivities

wi=∂XP∂pi⋅piXP
 MathType@MTEF@5@5@+=feaafiart1ev1aaatCvAUfKttLearuWrP9MDH5MBPbIqV92AaeXatLxBI9gBaebbnrfifHhDYfgasaacH8akY=wiFfYdH8Gipec8Eeeu0xXdbba9frFj0=OqFfea0dXdd9vqai=hGuQ8kuc9pgc9s8qqaq=dirpe0xb9q8qiLsFr0=vr0=vr0dc8meaabaqaciaacaGaaeqabaqabeGadaaakeaacqWG3bWDdaWgaaWcbaGaemyAaKgabeaakiabg2da9maalaaabaGaeyOaIyRaemiwaGLaemiuaafabaGaeyOaIyRaemiCaa3aaSbaaSqaaiabdMgaPbqabaaaaOGaeyyXIC9aaSaaaeaacqWGWbaCdaWgaaWcbaGaemyAaKgabeaaaOqaaiabdIfayjabdcfaqbaaaaa@40A8@

based on the sensitivity matrix *W *with

W=∑j(wiTwi)|j
 MathType@MTEF@5@5@+=feaafiart1ev1aaatCvAUfKttLearuWrP9MDH5MBPbIqV92AaeXatLxBI9gBaebbnrfifHhDYfgasaacH8akY=wiFfYdH8Gipec8Eeeu0xXdbba9frFj0=OqFfea0dXdd9vqai=hGuQ8kuc9pgc9s8qqaq=dirpe0xb9q8qiLsFr0=vr0=vr0dc8meaabaqaciaacaGaaeqabaqabeGadaaakeaacqWGxbWvcqGH9aqpdaaeqbqaaiabcIcaOiabdEha3naaDaaaleaacqWGPbqAaeaacqWGubavaaGccqWG3bWDdaWgaaWcbaGaemyAaKgabeaakiabcMcaPiabcYha8naaBaaaleaacqWGQbGAaeqaaaqaaiabdQgaQbqab0GaeyyeIuoaaaa@3E5B@

with *j *is the index of the simulated data points was calculated. Together with a constraint, considering the deflection of the parameters Δp¯
 MathType@MTEF@5@5@+=feaafiart1ev1aaatCvAUfKttLearuWrP9MDH5MBPbIqV92AaeXatLxBI9gBaebbnrfifHhDYfgasaacH8akY=wiFfYdH8Gipec8Eeeu0xXdbba9frFj0=OqFfea0dXdd9vqai=hGuQ8kuc9pgc9s8qqaq=dirpe0xb9q8qiLsFr0=vr0=vr0dc8meaabaqaciaacaGaaeqabaqabeGadaaakeaadaadaaqaaiabfs5aejabdchaWbaaaaa@2F8B@

**Figure 4:**
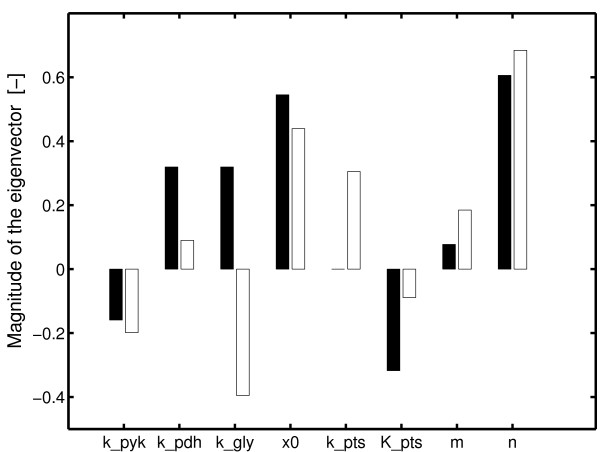
Results of the sensitivity analysis. Black bars indicate a non-PTS substrate while white bars indicate a PTS substrate. The size of the bars represent the level of the eigenvector of the sensitivity matrix *W *that correspond to the maximal eigenvalue. Note, that for the non-PTS case, parameter *k*_*pts *_has zero sensitivity (*r*_*pts *_is zero in this case).

Δp¯T⋅Δp¯=1
 MathType@MTEF@5@5@+=feaafiart1ev1aaatCvAUfKttLearuWrP9MDH5MBPbIqV92AaeXatLxBI9gBaebbnrfifHhDYfgasaacH8akY=wiFfYdH8Gipec8Eeeu0xXdbba9frFj0=OqFfea0dXdd9vqai=hGuQ8kuc9pgc9s8qqaq=dirpe0xb9q8qiLsFr0=vr0=vr0dc8meaabaqaciaacaGaaeqabaqabeGadaaakeaadaadaaqaaiabfs5aejabdchaWbaadaahaaWcbeqaaiabdsfaubaakiabgwSixpaamaaabaGaeuiLdqKaemiCaahaaiabg2da9iabigdaXaaa@3812@

the maximal deviation of the trajectories can be calculated by the eigenvectors and eigenvalues of matrix *W *[9]. The eigenvector corresponding to the maximal eigenvalue is Δp¯max
 MathType@MTEF@5@5@+=feaafiart1ev1aaatCvAUfKttLearuWrP9MDH5MBPbIqV92AaeXatLxBI9gBaebbnrfifHhDYfgasaacH8akY=wiFfYdH8Gipec8Eeeu0xXdbba9frFj0=OqFfea0dXdd9vqai=hGuQ8kuc9pgc9s8qqaq=dirpe0xb9q8qiLsFr0=vr0=vr0dc8meaabaqaciaacaGaaeqabaqabeGadaaakeaadaadaaqaaiabfs5aejabdchaWbaadaahaaWcbeqaaiabd2gaTjabdggaHjabdIha4baaaaa@33DF@. The parameter vector that leads to the maximal deviation is calculated then by *p* (1 + Δp¯max
 MathType@MTEF@5@5@+=feaafiart1ev1aaatCvAUfKttLearuWrP9MDH5MBPbIqV92AaeXatLxBI9gBaebbnrfifHhDYfgasaacH8akY=wiFfYdH8Gipec8Eeeu0xXdbba9frFj0=OqFfea0dXdd9vqai=hGuQ8kuc9pgc9s8qqaq=dirpe0xb9q8qiLsFr0=vr0=vr0dc8meaabaqaciaacaGaaeqabaqabeGadaaakeaadaadaaqaaiabfs5aejabdchaWbaadaahaaWcbeqaaiabd2gaTjabdggaHjabdIha4baaaaa@33DF@). Figure 4 summarizes the results. Four of the parameters are related to enzyme concentrations (*X*_0_, *k*_*gly*_, *k*_*pyk*_, *k*_*pdh*_) while the others are kinetic parameters of the PTS reaction (*k*_*pts*_, *K*_*pts*_) and the pyruvate kinase reaction (*m*, *n*). Interestingly, in the kinetic expression *f *of the pyruvate kinase parameter *n *describing the influence of the feed-forward control (activation of the pyruvate kinase by glucose 6-phosphate) shows maximal sensitivity in both cases. In general, the amount of enzyme has a bigger influence than the kinetic parameters. This will allow the cell to adjust the degree of phosphorylation by genetic control.

#### Gluconeogenetic substrates

For gluconeogentic substrates the scheme according to Figure 2, case B, is considered. The o.d.e's are:

P˙EP=h1rup+rpps−rpts−rglu
 MathType@MTEF@5@5@+=feaafiart1ev1aaatCvAUfKttLearuWrP9MDH5MBPbIqV92AaeXatLxBI9gBaebbnrfifHhDYfgasaacH8akY=wiFfYdH8Gipec8Eeeu0xXdbba9frFj0=OqFfea0dXdd9vqai=hGuQ8kuc9pgc9s8qqaq=dirpe0xb9q8qiLsFr0=vr0=vr0dc8meaabaqaciaacaGaaeqabaqabeGadaaakeaacuWGqbaugaGaaiabdweafjabdcfaqjabg2da9iabdIgaOnaaBaaaleaacqaIXaqmaeqaaOGaemOCai3aaSbaaSqaaiabdwha1jabdchaWbqabaGccqGHRaWkcqWGYbGCdaWgaaWcbaGaemiCaaNaemiCaaNaem4CamhabeaakiabgkHiTiabdkhaYnaaBaaaleaacqWGWbaCcqWG0baDcqWGZbWCaeqaaOGaeyOeI0IaemOCai3aaSbaaSqaaiabdEgaNjabdYgaSjabdwha1bqabaaaaa@4C6E@

P˙rv=h2rup+rpts−rpps−rbio
 MathType@MTEF@5@5@+=feaafiart1ev1aaatCvAUfKttLearuWrP9MDH5MBPbIqV92AaeXatLxBI9gBaebbnrfifHhDYfgasaacH8akY=wiFfYdH8Gipec8Eeeu0xXdbba9frFj0=OqFfea0dXdd9vqai=hGuQ8kuc9pgc9s8qqaq=dirpe0xb9q8qiLsFr0=vr0=vr0dc8meaabaqaciaacaGaaeqabaqabeGadaaakeaacuWGqbaugaGaaiabdkhaYjabdAha2jabg2da9iabdIgaOnaaBaaaleaacqaIYaGmaeqaaOGaemOCai3aaSbaaSqaaiabdwha1jabdchaWbqabaGccqGHRaWkcqWGYbGCdaWgaaWcbaGaemiCaaNaemiDaqNaem4CamhabeaakiabgkHiTiabdkhaYnaaBaaaleaacqWGWbaCcqWGWbaCcqWGZbWCaeqaaOGaeyOeI0IaemOCai3aaSbaaSqaaiabdkgaIjabdMgaPjabd+gaVbqabaaaaa@4CFA@

X˙P=rpts.
 MathType@MTEF@5@5@+=feaafiart1ev1aaatCvAUfKttLearuWrP9MDH5MBPbIqV92AaeXatLxBI9gBaebbnrfifHhDYfgasaacH8akY=wiFfYdH8Gipec8Eeeu0xXdbba9frFj0=OqFfea0dXdd9vqai=hGuQ8kuc9pgc9s8qqaq=dirpe0xb9q8qiLsFr0=vr0=vr0dc8meaabaqaciaacaGaaeqabaqabeGadaaakeaacuWGybawgaGaaiabdcfaqjabg2da9iabdkhaYnaaBaaaleaacqWGWbaCcqWG0baDcqWGZbWCaeqaaOGaeiOla4caaa@36ED@

Rate *r*_*up *_is the system input. Rate *r*_*bio *_is the flux from pyruvate to biosynthesis and *r*_*glu *_is the rate of gluconeogenesis:

*r*_*bio *_= *k*_*bio*_*P*_*rv*_

*r*_*glu *_= *k*_*glu*_*PEP*

For the rate *r*_*pps *_the following simple approach is used:

*r*_*pps *_= *k*_*pps*_*Prv g*(*Prv*, ...)

with function *g *representing the influence of pyruvate and possible effectors. Together with parameters *h*_1_, *h*_2 _and *k*_*pps *_the rates are adjusted in such a way that data from a flux distribution [10] can be described.The percentage fluxes can be found in Figure 2. The steady-state equation for *XP *can be rewritten as:

XP=X01+KptsPrvPEP.
 MathType@MTEF@5@5@+=feaafiart1ev1aaatCvAUfKttLearuWrP9MDH5MBPbIqV92AaeXatLxBI9gBaebbnrfifHhDYfgasaacH8akY=wiFfYdH8Gipec8Eeeu0xXdbba9frFj0=OqFfea0dXdd9vqai=hGuQ8kuc9pgc9s8qqaq=dirpe0xb9q8qiLsFr0=vr0=vr0dc8meaabaqaciaacaGaaeqabaqabeGadaaakeaacqWGybawcqWGqbaucqGH9aqpdaWcaaqaaiabdIfaynaaBaaaleaacqaIWaamaeqaaaGcbaGaeGymaeJaey4kaSIaem4saS0aaSbaaSqaaiabdchaWjabdsha0jabdohaZbqabaGcdaWcaaqaaiabdcfaqjabdkhaYjabdAha2bqaaiabdcfaqjabdweafjabdcfaqbaaaaGaeiOla4caaa@4255@

**Figure 5:**
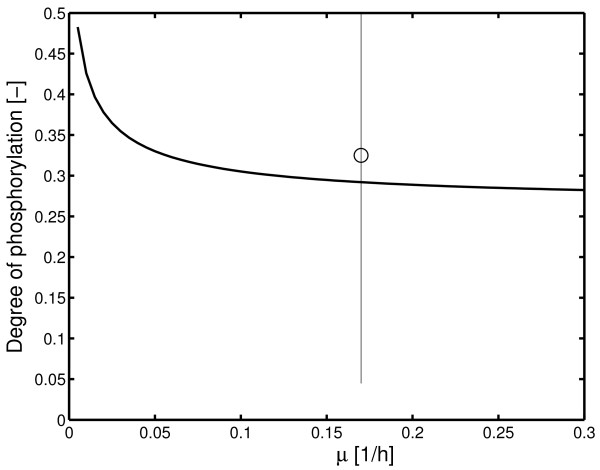
Degree of phosphorylation in dependence on the growth on acetate. Measurements are available for four experiments with nearly identical growth rate (errors bar is given for the four experiments). and the mean value is plotted.

Simulation studies lead to the conclusion that function *g *should depend on *PEP *that acts as an inhibitor of PEP synthase. Otherwise, the degree of phosphorylation increases with increasing uptake rate which seems, also in this case, not to be meaningful. Indeed, literature research revealed that PEP synthase is negatively regulated by PEP [11]. Function *g *used is:

g=g1(PEP)⋅g2(Prv)=1PEP2⋅Prv,
 MathType@MTEF@5@5@+=feaafiart1ev1aaatCvAUfKttLearuWrP9MDH5MBPbIqV92AaeXatLxBI9gBaebbnrfifHhDYfgasaacH8akY=wiFfYdH8Gipec8Eeeu0xXdbba9frFj0=OqFfea0dXdd9vqai=hGuQ8kuc9pgc9s8qqaq=dirpe0xb9q8qiLsFr0=vr0=vr0dc8meaabaqaciaacaGaaeqabaqabeGadaaakeaacqWGNbWzcqGH9aqpcqWGNbWzdaWgaaWcbaGaeGymaedabeaakiabcIcaOiabdcfaqjabdweafjabdcfaqjabcMcaPiabgwSixlabdEgaNnaaBaaaleaacqaIYaGmaeqaaOGaeiikaGIaemiuaaLaemOCaiNaemODayNaeiykaKIaeyypa0ZaaSaaaeaacqaIXaqmaeaacqWGqbaucqWGfbqrcqWGqbaudaahaaWcbeqaaiabikdaYaaaaaGccqGHflY1cqWGqbaucqWGYbGCcqWG2bGDcqGGSaalaaa@4EEC@

taking into account that Pps is a dimer with two possible binding sites. A simulation study for different values of acetate uptake/growth rates are shown in Figure 5; data are taken from Table 3 [Additional file 1]. Another interesting observation where the PEP/pyruvate ratio may be involved was reported by the group of Liao [12]. They analyzed a wild type strain and a *pps *mutant strain when glucose and acetate are provided in the medium. They showed that the missing Pps protein has no influence on the general physiology but shows a significant influence on the transition time from growth on glucose to growth on acetate. In this case the degree of phosphorylation is a constant value:

XP=X01+Kptsh2kgluh1kbio.
 MathType@MTEF@5@5@+=feaafiart1ev1aaatCvAUfKttLearuWrP9MDH5MBPbIqV92AaeXatLxBI9gBaebbnrfifHhDYfgasaacH8akY=wiFfYdH8Gipec8Eeeu0xXdbba9frFj0=OqFfea0dXdd9vqai=hGuQ8kuc9pgc9s8qqaq=dirpe0xb9q8qiLsFr0=vr0=vr0dc8meaabaqaciaacaGaaeqabaqabeGadaaakeaacqWGybawcqWGqbaucqGH9aqpdaWcaaqaaiabdIfaynaaBaaaleaacqaIWaamaeqaaaGcbaGaeGymaeJaey4kaSIaem4saS0aaSbaaSqaaiabdchaWjabdsha0jabdohaZbqabaGcdaWcaaqaaiabdIgaOnaaBaaaleaacqaIYaGmaeqaaOGaem4AaS2aaSbaaSqaaiabdEgaNjabdYgaSjabdwha1bqabaaakeaacqWGObaAdaWgaaWcbaGaeGymaedabeaakiabdUgaRnaaBaaaleaacqWGIbGycqWGPbqAcqWGVbWBaeqaaaaaaaGccqGGUaGlaaa@4B49@

Liao and colleagues observed a drastic increase of the lag phase on acetate in the mutant strain during glucose/acetate diauxic growth. Our simple model predicts, that the degree of phosphorylation is a bit smaller than the values in the wild type strain. This confirms that Pps has nearly no influence on physiological parameters like the growth rate.

### Model predictions

With the model developed so far, model predictions can be performed. Two cases are considered: the PEP/pyruvate ratio and growth on different single carbon sources.

#### PEP/pyruvate ratio

**Figure 6:**
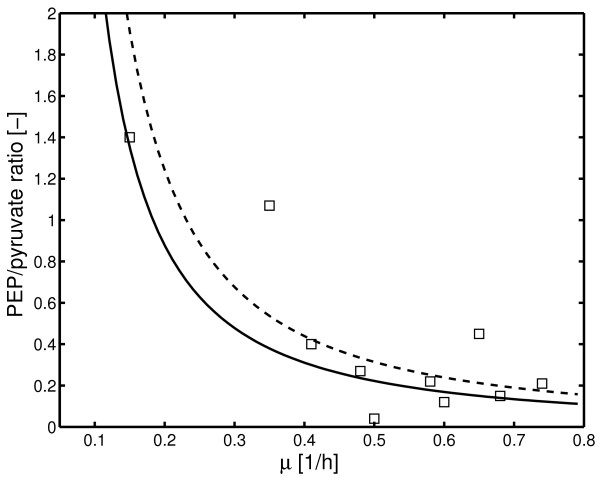
Measured and simulated relationship between the PEP/pyruvate ratio and the specific growth rate. Solid line is for PTS carbohydrates and the dashed line for non-PTS carbohydrates. Symbols respresent measured values (Table 3 in [Additional file 1] [4]).

The PEP/pyruvate ratio could be predicted in dependency on the growth rate. Experimental data were taken from [4] and compared to the simulation results (Figure 6). As can be seen, the prediction fits to the data well.

#### Growth on single carbohydrates

**Figure 7:**
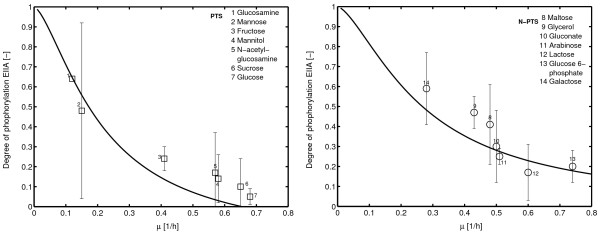
Experimental data showing the relationship between the specific growth rate *μ *and the degree of phosphorylation of EIIA^*Crr *^for a number of different experiments performed with single carbohydrates. Left: PTS carbohydrates as indicated in the legend. Right: non-PTS carbohydrates as indicated in the legend. Samples are taken in the mid-log phase. Error bars indicate a 95% confidence interval.

To confirm that the model presented here is able to describe the sensor system for a number of carbohydrates, experimental data from batch experiments with different PTS and non-PTS carbohydrates were performed and compared with the model calculations [4]. As can be seen in Figure 7 the experimental results are in good agreement for a number of substrates. Except for N-acetyl-glucosamine, the measured data points fit well to the prediction. Note, that most of the PTS sugars use the phosphoryl group from HPr to transport the carbohydrate into the cell. If this is included in the calculation, the degree of phosphorylation of EIIA^*Crr *^*d*_*EIIA *_depends on the fraction of phosphorylated HPr *d*_*HPr*_:

dEIIA=EIIAPEIIA0=dHPrdHPr+K3(1−dHPr)
 MathType@MTEF@5@5@+=feaafiart1ev1aaatCvAUfKttLearuWrP9MDH5MBPbIqV92AaeXatLxBI9gBaebbnrfifHhDYfgasaacH8akY=wiFfYdH8Gipec8Eeeu0xXdbba9frFj0=OqFfea0dXdd9vqai=hGuQ8kuc9pgc9s8qqaq=dirpe0xb9q8qiLsFr0=vr0=vr0dc8meaabaqaciaacaGaaeqabaqabeGadaaakeaacqWGKbazdaWgaaWcbaGaemyrauKaemysaKKaemysaKKaemyqaeeabeaakiabg2da9maalaaabaGaemyrauKaemysaKKaemysaKKaemyqae0aaWbaaSqabeaacqWGqbauaaaakeaacqWGfbqrcqWGjbqscqWGjbqscqWGbbqqdaWgaaWcbaGaeGimaadabeaaaaGccqGH9aqpdaWcaaqaaiabdsgaKnaaBaaaleaacqWGibasieGacqWFqbaucqWFYbGCaeqaaaGcbaGaemizaq2aaSbaaSqaaiabdIeaijab=bfaqjab=jhaYbqabaGccqGHRaWkcqWGlbWsdaWgaaWcbaGaeG4mamdabeaakiabcIcaOiabigdaXiabgkHiTiabdsgaKnaaBaaaleaacqWGibascqWFqbaucqWFYbGCaeqaaOGaeiykaKcaaaaa@562E@

Since the value for *K*_3_, the equilibrium constant for the phosphoryl transfer HPr to EIIA^*Crr *^is approximately 1 [3,13,14], values of *d*_*EIIA *_and *d*_*HPr *_are nearly equal. Therefore, in the model, state variable *X *can be used to represent HPr as well as EIIA^*Crr*^.

### Transcription efficiency and sensor kinetics

In order to set up a closed loop, further modules have to be characterized. First, the influence of phosphorylated EIIA^*Crr *^on transcription efficiency is analyzed, afterwards the kinetics of the PTS transport system is investigated.

#### Transcription efficiency

**Figure 8:**
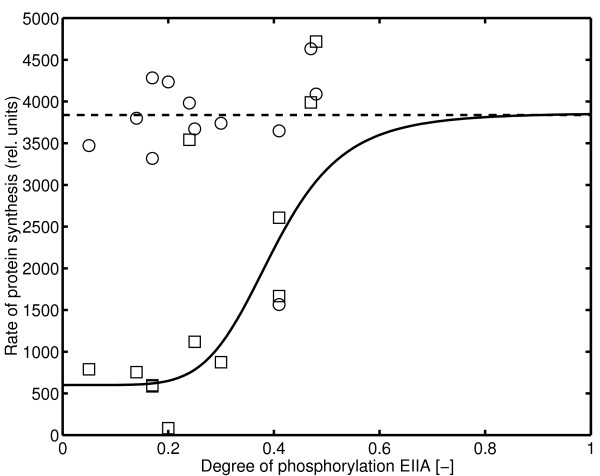
Relationship between the degree of phosphorylation of EIIA^*Crr*^and promoter activity of *scrY *(squares, dependent from transcription factor Crp) and *scrK *(circles, independent from transcription factor Crp) [4]. The activity of the reporter protein is taken as a measure for protein synthesis. To calculate the transcription efficiency the raw data are multiplied with the specific growth rate *μ*. (Data from Table 3 in [Additional file 1] [4]).

Experiments to determine the influence of the degree of phosphorylation of EIIA^*Crr *^on the transcription efficiency were performed with the cAMP·Crp independent promoter *scrK*_*P *_and the cAMP·Crp dependent promoter *scrY*_*P *_[4]. As can be seen in Figure 8, the activity of the cAMP·Crp independent promoter does not vary with the degree of phosphorylation of EIIA^*Crr *^while the cAMP·Crp dependent promoter shows a sigmoidal behavior in the range below 0.6. From the data, a sigmoidal function *g*_*T *_could be determined that maps the degree of phosphorylation of EIIA^*Crr *^to the rate of protein synthesis:

gT=kb+ksynXP6XP6+K6.
 MathType@MTEF@5@5@+=feaafiart1ev1aaatCvAUfKttLearuWrP9MDH5MBPbIqV92AaeXatLxBI9gBaebbnrfifHhDYfgasaacH8akY=wiFfYdH8Gipec8Eeeu0xXdbba9frFj0=OqFfea0dXdd9vqai=hGuQ8kuc9pgc9s8qqaq=dirpe0xb9q8qiLsFr0=vr0=vr0dc8meaabaqaciaacaGaaeqabaqabeGadaaakeaacqWGNbWzdaWgaaWcbaGaemivaqfabeaakiabg2da9iabdUgaRnaaBaaaleaacqWGIbGyaeqaaOGaey4kaSIaem4AaS2aaSbaaSqaaiabdohaZjabdMha5jabd6gaUbqabaGcdaWcaaqaaiabdIfayjabdcfaqnaaCaaaleqabaGaeGOnaydaaaGcbaGaemiwaGLaemiuaa1aaWbaaSqabeaacqaI2aGnaaGccqGHRaWkcqWGlbWsdaahaaWcbeqaaiabiAda2aaaaaGccqGGUaGlaaa@4564@

Unexpectedly, the Hill coefficient is high (*n *= 6) indicating a high sensitivity in a narrow range of the input.

#### Sensor kinetics

**Figure 9:**
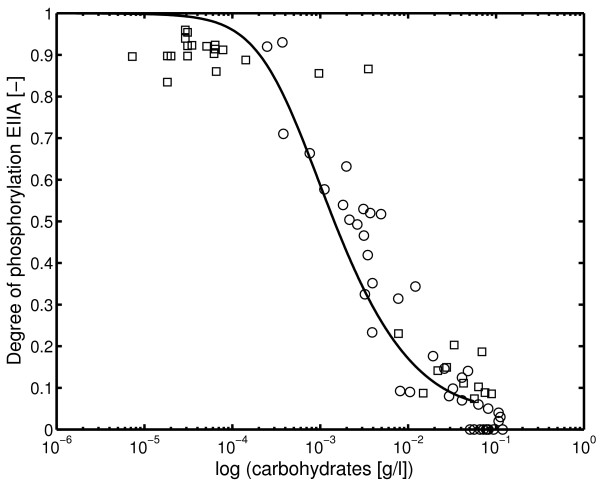
Measured and simulated relationship between residual carbohydrate concentrations during a continuous bioreactor experiments and the measured degree of phosphorylation of protein EIIA^*Crr*^. Values are given for glucose (circles) and mannitol (squares). See Table 4 and 5 in [Additional file 1].

Experiments to determine the apparent *K*_*M *_value of the PTS transporter for different PTS carbohydrates are reported in a number of publications [15]. In [4] experimental data determining the phosphorylation levels near these critical substrate concentrations are taken during continuous bioreactor experiments. During the starting phase of the continuous bioreactor experiments, the carbohydrate concentration drops until it becomes limiting. This decrease is much slower than it is in batch experiments, allowing for a better resolution of data in the low carbohydrate concentration ranges. Experiments were performed with the PTS substrates glucose and mannitol, having similar *K*_*M *_values as determined in transport assays. To determine the kinetic parameters of the PTS, a two-substrate kinetics of the form

rpts_up=kpts⋅upEGlcGlc XP(Glc+Kglc)(XP+KEIIAP)
 MathType@MTEF@5@5@+=feaafiart1ev1aaatCvAUfKttLearuWrP9MDH5MBPbIqV92AaeXatLxBI9gBaebbnrfifHhDYfgasaacH8akY=wiFfYdH8Gipec8Eeeu0xXdbba9frFj0=OqFfea0dXdd9vqai=hGuQ8kuc9pgc9s8qqaq=dirpe0xb9q8qiLsFr0=vr0=vr0dc8meaabaqaciaacaGaaeqabaqabeGadaaakeaacqWGYbGCdaWgaaWcbaGaemiCaaNaemiDaqNaem4CamNaei4xa8LaemyDauNaemiCaahabeaakiabg2da9iabdUgaRnaaBaaaleaacqWGWbaCcqWG0baDcqWGZbWCcqGHflY1cqWG1bqDcqWGWbaCaeqaaOGaemyrau0aaSbaaSqaaiabdEeahjabdYgaSjabdogaJbqabaGcdaWcaaqaaiabdEeahjabdYgaSjabdogaJjabbccaGiabdIfayjabdcfaqbqaaiabcIcaOiabdEeahjabdYgaSjabdogaJjabgUcaRiabdUealnaaBaaaleaacqWGNbWzcqWGSbaBcqWGJbWyaeqaaOGaeiykaKIaeiikaGIaemiwaGLaemiuaaLaey4kaSIaem4saS0aaSbaaSqaaiabdweafjabdMeajjabdMeajjabdgeabjabdcfaqbqabaGccqGGPaqkaaaaaa@6653@

with enzyme concentration *E*_*Glc*_, turnover number *k*_*pts*·*up *_and binding constants *K*_*i *_is used. The parameters are determined from the dynamical experiments and are compared with the experimental data (for experimental data, see Tables in [Additional file 1]; for parameter values, see Table 6 in [Additional file 1]). Figure 9 shows the relationship between the measured residual carbohydrate concentrations during the bioreactor experiments and the measured degree of phosphorylation of protein EIIA^*Crr *^together with a simulation result.

### Closed loop dynamics and application to diauxic growth

Finally, a model with a closed loop, comprising the core model and individual uptake systems is set up. The model is applied to a complex growth situation, namely growth with a mixture of two substrates. Simulation studies for growth on mixtures of glucose/glucose 6-phosphate and of glucose/lactose are described.

#### Growth on glucose/glucose 6-phosphate

Glucose 6-phosphate represents an interesting growth substrate. This sugar-phosphate is taken up into the cell via the inorganic phosphate antiporter, UhpT [16] and can afterwards enter into glycolysis without further modification. A mixture of glucose and glucose 6-phosphate is a very interesting case because expression of both proteins depends on the cAMP·CRP complex. UhpT has been shown to influence cAMP levels in the cell [17]. It was concluded that neither glucose 6-phosphate nor another metabolite of glycolysis was directly involved in this effect but rather the flux through UhpT itself [17]. These results are confirmed by additional studies analyzing the effect of glucose 6-phosphate uptake on the degree of EIIA^*Crr *^phosphorylation and the amount of cAMP [18]. In addition, it was shown that high intracellular Glc6P levels lead to the degradation of the *ptsG *mRNA [6,7] via the small regulatory RNA, SgrS [5] and hence to reduced concentrations of PtsG.

**Figure 10:**
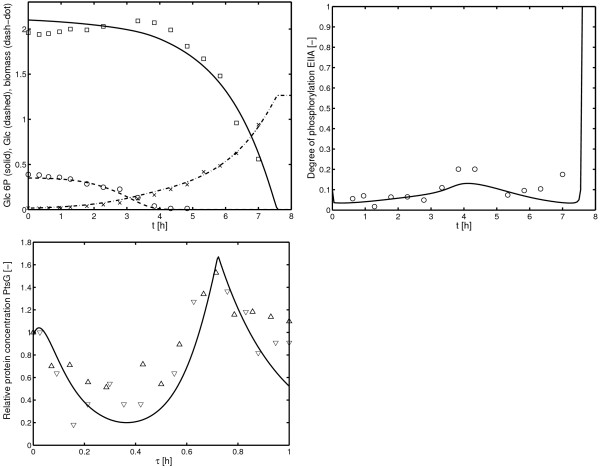
Glucose 6phosphate/glucose diauxic growth. Top: Time course of glucose 6-phosphate, glucose and biomass. Middle: Time course of the degree of phosphorylation of EIIA^*Crr*^. Bottom: Time course of the activity of the glucose transporter monitored by a reporter gene fusion (strain LZ110). Shown are two different experiments (symbols ∇ for experiment 1 and Δ for experiment 2); here, the time was scaled to the maximal time of the experiment (7 h and 7.6 h).

Carbohydrate transporters are inducible, that is, the enzymes are synthesized only if the respective substrate is present in the medium. To take this into account the rate of synthesis depends on Equation (37) and a second term gIglc
 MathType@MTEF@5@5@+=feaafiart1ev1aaatCvAUfKttLearuWrP9MDH5MBPbIqV92AaeXatLxBI9gBaebbnrfifHhDYfgasaacH8akY=wiFfYdH8Gipec8Eeeu0xXdbba9frFj0=OqFfea0dXdd9vqai=hGuQ8kuc9pgc9s8qqaq=dirpe0xb9q8qiLsFr0=vr0=vr0dc8meaabaqaciaacaGaaeqabaqabeGadaaakeaacqWGNbWzdaqhaaWcbaGaemysaKeabaGaem4zaCMaemiBaWMaem4yamgaaaaa@3352@ and gIg6p
 MathType@MTEF@5@5@+=feaafiart1ev1aaatCvAUfKttLearuWrP9MDH5MBPbIqV92AaeXatLxBI9gBaebbnrfifHhDYfgasaacH8akY=wiFfYdH8Gipec8Eeeu0xXdbba9frFj0=OqFfea0dXdd9vqai=hGuQ8kuc9pgc9s8qqaq=dirpe0xb9q8qiLsFr0=vr0=vr0dc8meaabaqaciaacaGaaeqabaqabeGadaaakeaacqWGNbWzdaqhaaWcbaGaemysaKeabaGaem4zaCMaeGOnayJaemiCaahaaaaa@3305@, respectively, that describes induction. Although, it is known that high levels of glucose 6-phosphate influence glucose uptake, in the model, no interaction of glucose 6-phosphate as inhibitor of the transporter is included, since quantitative data are hardly available (see discussion on this topic in [17]). However, as can be seen in Figure 10 a strong inhibition of glucose uptake and concomitant, a decrease of the amount of the PtsG transporter is observed during glucose 6-phosphate uptake. To match these unexpected experimental data, an influence of the glucose 6-phosphate uptake system (*E*_*G*6*p*_) on the rate of synthesis rsynglc
 MathType@MTEF@5@5@+=feaafiart1ev1aaatCvAUfKttLearuWrP9MDH5MBPbIqV92AaeXatLxBI9gBaebbnrfifHhDYfgasaacH8akY=wiFfYdH8Gipec8Eeeu0xXdbba9frFj0=OqFfea0dXdd9vqai=hGuQ8kuc9pgc9s8qqaq=dirpe0xb9q8qiLsFr0=vr0=vr0dc8meaabaqaciaacaGaaeqabaqabeGadaaakeaacqWGYbGCdaqhaaWcbaGaem4CamNaemyEaKNaemOBa4gabaGaem4zaCMaemiBaWMaem4yamgaaaaa@369C@ of the glucose transporter is formulated as a "black box" model, function *g*_*B *_(Equation (43)). This is done to account for possible effects on *ptsG *mRNA stability. The model introduced so far is complemented with equations for the substrates *Glc*6*P*, *Glc*, biomass *B*, and kinetics for the glucose 6-phosphate uptake. The additional equations are:

B˙=μ B
 MathType@MTEF@5@5@+=feaafiart1ev1aaatCvAUfKttLearuWrP9MDH5MBPbIqV92AaeXatLxBI9gBaebbnrfifHhDYfgasaacH8akY=wiFfYdH8Gipec8Eeeu0xXdbba9frFj0=OqFfea0dXdd9vqai=hGuQ8kuc9pgc9s8qqaq=dirpe0xb9q8qiLsFr0=vr0=vr0dc8meaabaqaciaacaGaaeqabaqabeGadaaakeaacuWGcbGqgaGaaiabg2da9GGaciab=X7aTjabbccaGiabdkeacbaa@3259@

Glc˙6P=−rn−ptsB=−kg6pEg6pGlc6PGlc6P+Kg6pB
 MathType@MTEF@5@5@+=feaafiart1ev1aaatCvAUfKttLearuWrP9MDH5MBPbIqV92AaeXatLxBI9gBaebbnrfifHhDYfgasaacH8akY=wiFfYdH8Gipec8Eeeu0xXdbba9frFj0=OqFfea0dXdd9vqai=hGuQ8kuc9pgc9s8qqaq=dirpe0xb9q8qiLsFr0=vr0=vr0dc8meaabaqaciaacaGaaeqabaqabeGadaaakeaacqWGhbWrcqWGSbaBcuWGJbWygaGaaiabiAda2iabdcfaqjabg2da9iabgkHiTiabdkhaYnaaBaaaleaacqWGUbGBcqGHsislcqWGWbaCcqWG0baDcqWGZbWCaeqaaOGaemOqaiKaeyypa0JaeyOeI0Iaem4AaS2aaSbaaSqaaiabdEgaNjabiAda2iabdchaWbqabaGccqWGfbqrdaWgaaWcbaGaem4zaCMaeGOnayJaemiCaahabeaakmaalaaabaGaem4raCKaemiBaWMaem4yamMaeGOnayJaemiuaafabaGaem4raCKaemiBaWMaem4yamMaeGOnayJaemiuaaLaey4kaSIaem4saS0aaSbaaSqaaiabdEgaNjabiAda2iabdchaWbqabaaaaOGaemOqaieaaa@5D04@

G˙lc=−rpts_upB
 MathType@MTEF@5@5@+=feaafiart1ev1aaatCvAUfKttLearuWrP9MDH5MBPbIqV92AaeXatLxBI9gBaebbnrfifHhDYfgasaacH8akY=wiFfYdH8Gipec8Eeeu0xXdbba9frFj0=OqFfea0dXdd9vqai=hGuQ8kuc9pgc9s8qqaq=dirpe0xb9q8qiLsFr0=vr0=vr0dc8meaabaqaciaacaGaaeqabaqabeGadaaakeaacuWGhbWrgaGaaiabdYgaSjabdogaJjabg2da9iabgkHiTiabdkhaYnaaBaaaleaacqWGWbaCcqWG0baDcqWGZbWCcqGGFbWxcqWG1bqDcqWGWbaCaeqaaOGaemOqaieaaa@3D8A@

E˙G6p=rsyng6p−(μ+kd)Eg6p=k1gIg6pgT−(μ+kd)EG6p=k1rn−ptsrn−pts+K1gT−μ EG6p
 MathType@MTEF@5@5@+=feaafiart1ev1aaatCvAUfKttLearuWrP9MDH5MBPbIqV92AaeXatLxBI9gBaebbnrfifHhDYfgasaacH8akY=wiFfYdH8Gipec8Eeeu0xXdbba9frFj0=OqFfea0dXdd9vqai=hGuQ8kuc9pgc9s8qqaq=dirpe0xb9q8qiLsFr0=vr0=vr0dc8meaabaqaciaacaGaaeqabaqabeGadaaakeaafaqaaeGadaaabaGafmyrauKbaiaadaWgaaWcbaGaem4raCKaeGOnayJaemiCaahabeaaaOqaaiabg2da9aqaaiabdkhaYnaaDaaaleaacqWGZbWCcqWG5bqEcqWGUbGBaeaacqWGNbWzcqaI2aGncqWGWbaCaaGccqGHsislcqGGOaakiiGacqWF8oqBcqGHRaWkcqWGRbWAdaWgaaWcbaGaemizaqgabeaakiabcMcaPiabdweafnaaBaaaleaacqWGNbWzcqaI2aGncqWGWbaCaeqaaOGaeyypa0Jaem4AaS2aaSbaaSqaaiabigdaXaqabaGccqWGNbWzdaqhaaWcbaGaemysaKeabaGaem4zaCMaeGOnayJaemiCaahaaOGaem4zaC2aaSbaaSqaaiabdsfaubqabaGccqGHsislcqGGOaakcqWF8oqBcqGHRaWkcqWGRbWAdaWgaaWcbaGaemizaqgabeaakiabcMcaPiabdweafnaaBaaaleaacqWGhbWrcqaI2aGncqWGWbaCaeqaaaGcbaaabaGaeyypa0dabaGaem4AaS2aaSbaaSqaaiabigdaXaqabaGcdaWcaaqaaiabdkhaYnaaBaaaleaacqWGUbGBcqGHsislcqWGWbaCcqWG0baDcqWGZbWCaeqaaaGcbaGaemOCai3aaSbaaSqaaiabd6gaUjabgkHiTiabdchaWjabdsha0jabdohaZbqabaGccqGHRaWkcqWGlbWsdaWgaaWcbaGaeGymaedabeaaaaGccqWGNbWzdaWgaaWcbaGaemivaqfabeaakiabgkHiTiab=X7aTjabbccaGiabdweafnaaBaaaleaacqWGhbWrcqaI2aGncqWGWbaCaeqaaaaaaaa@8505@

E˙Glc=rsynglc−(μ+kd)EGlc=k2gIglcgB gT−(μ+kd)EGlc=k2rptsrpts+K2KIKI+EG6pgT−(μ+kd)EGlc,
 MathType@MTEF@5@5@+=feaafiart1ev1aaatCvAUfKttLearuWrP9MDH5MBPbIqV92AaeXatLxBI9gBaebbnrfifHhDYfgasaacH8akY=wiFfYdH8Gipec8Eeeu0xXdbba9frFj0=OqFfea0dXdd9vqai=hGuQ8kuc9pgc9s8qqaq=dirpe0xb9q8qiLsFr0=vr0=vr0dc8meaabaqaciaacaGaaeqabaqabeGadaaakeaafaqaaeGadaaabaGafmyrauKbaiaadaWgaaWcbaGaem4raCKaemiBaWMaem4yamgabeaaaOqaaiabg2da9aqaaiabdkhaYnaaDaaaleaacqWGZbWCcqWG5bqEcqWGUbGBaeaacqWGNbWzcqWGSbaBcqWGJbWyaaGccqGHsislcqGGOaakiiGacqWF8oqBcqGHRaWkcqWGRbWAdaWgaaWcbaGaemizaqgabeaakiabcMcaPiabdweafnaaBaaaleaacqWGhbWrcqWGSbaBcqWGJbWyaeqaaOGaeyypa0Jaem4AaS2aaSbaaSqaaiabikdaYaqabaGccqWGNbWzdaqhaaWcbaGaemysaKeabaGaem4zaCMaemiBaWMaem4yamgaaOGaem4zaC2aaSbaaSqaaiabdkeacbqabaGccqqGGaaicqWGNbWzdaWgaaWcbaGaemivaqfabeaakiabgkHiTiabcIcaOiab=X7aTjabgUcaRiabdUgaRnaaBaaaleaacqWGKbazaeqaaOGaeiykaKIaemyrau0aaSbaaSqaaiabdEeahjabdYgaSjabdogaJbqabaaakeaaaeaacqGH9aqpaeaacqWGRbWAdaWgaaWcbaGaeGOmaidabeaakmaalaaabaGaemOCai3aaSbaaSqaaiabdchaWjabdsha0jabdohaZbqabaaakeaacqWGYbGCdaWgaaWcbaGaemiCaaNaemiDaqNaem4CamhabeaakiabgUcaRiabdUealnaaBaaaleaacqaIYaGmaeqaaaaakmaalaaabaGaem4saS0aaSbaaSqaaiabdMeajbqabaaakeaacqWGlbWsdaWgaaWcbaGaemysaKeabeaakiabgUcaRiabdweafnaaBaaaleaacqWGhbWrcqaI2aGncqWGWbaCaeqaaaaakiabdEgaNnaaBaaaleaacqWGubavaeqaaOGaeyOeI0IaeiikaGIae8hVd0Maey4kaSIaem4AaS2aaSbaaSqaaiabdsgaKbqabaGccqGGPaqkcqWGfbqrdaWgaaWcbaGaem4raCKaemiBaWMaem4yamgabeaakiabcYcaSaaaaaa@9588@

with the specific growth rate *μ *that is calculated with the yield coefficients *Y*_*g*6*p *_and *Y*_*glc *_in dependence on the substrate uptake:

*μ *= *Y*_*g*6*p *_*r*_*n*-*pts *_+ *Y*_*glc *_*r*_*pts_up*_.

Parameters *k*_1 _and *k*_2 _are scaling factors and *g*_*T *_is taken from Equation (37).

In the simulation (Figure 10), only the parameters for the glucose 6-phosphate uptake and the inhibition of the glucose transporter PtsG by the glucose 6-phosphate transporter UhpT are fitted while all other parameters are kept as described in the previous sections. Therefore, the time course of the degree of phosphorylation of EIIA^*Crr *^is a prediction based on previous results. The time course of the substrates in the medium hints to an inhibition of glucose uptake during glucose 6-phosphate uptake. After consumption of glucose 6-phosphate, the growth rate slows down which results in a small increase of the degree of phosphorylation. During subsequent growth on glucose, the degree of phosphorylation of EIIA^*Crr *^is again very low. For the experiment shown in Figure 10 the course of the glucose transporter was not measured. Therefore, the right plot of Figure 10 shows data from experiments with slightly different initial conditions. To compare the results, the time of the simulation experiment and the time of the wet experiment are scaled. The time course of the glucose transporter indicates that indeed the rate of gene expression is under control and is inhibited during growth on glucose 6-phosphate.

**Figure 11:**
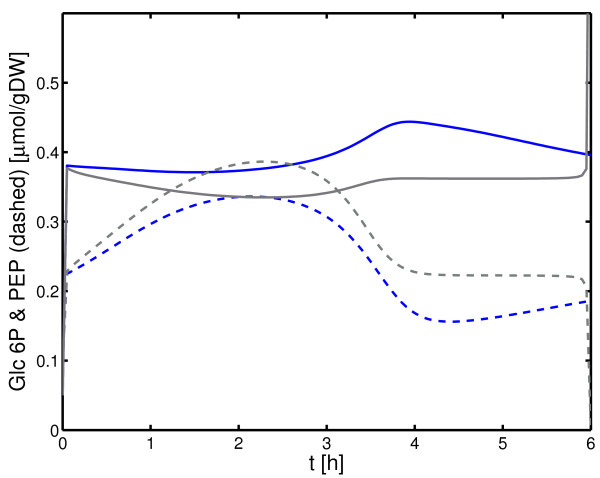
Simulation results for glucose 6-phosphate (solid lines) and PEP (dashed lines). The simulation compares two cases: the glucose transporter PtsG is under control of the glucose 6-phosphate transporter UhpT (black lines, corresponding to the simulation in Figure 9) or not (grey lines). Both simulation results in comparable concentrations of the two metabolites.

As described above, the cause for the down-regulation of PtsG is not clear. To check the intracellular levels of glycolytic metabolites, a simulation is performed that compare the experiment shown in Figure 10 with a model variant where no interaction between the two transporters is assumed (*K*_*I *_>> *E*_*G*6*p*_). As can be seen in Figure 11 the time course of glucose 6-phosphate and PEP are nearly equal in both experiments, indicating that these metabolites are hardly involved in the *ptsG *mRNA degradation.

#### Growth on glucose/lactose

Finally, we simulated a diauxic growth experiment with glucose and lactose already introduced in [3] with the reduced model introduced here. In the reduced model, gene expression is modeled with the characteristic curve for the relationship of the degree of phosphorylation of EIIA^*Crr *^on cAMP·Crp dependent promoters. The equation for the lactose in the medium, lactose transporter *E*_*Lac *_and for the transporter kinetics read:

La˙c=−rlac B=−klac ElacLacKlac+Lac(1+X0−XPKIEIIAX0)B
 MathType@MTEF@5@5@+=feaafiart1ev1aaatCvAUfKttLearuWrP9MDH5MBPbIqV92AaeXatLxBI9gBaebbnrfifHhDYfgasaacH8akY=wiFfYdH8Gipec8Eeeu0xXdbba9frFj0=OqFfea0dXdd9vqai=hGuQ8kuc9pgc9s8qqaq=dirpe0xb9q8qiLsFr0=vr0=vr0dc8meaabaqaciaacaGaaeqabaqabeGadaaakeaacqWGmbatcuWGHbqygaGaaiabdogaJjabg2da9iabgkHiTiabdkhaYnaaBaaaleaacqWGSbaBcqWGHbqycqWGJbWyaeqaaOGaeeiiaaIaemOqaiKaeyypa0JaeyOeI0Iaem4AaS2aaSbaaSqaaiabdYgaSjabdggaHjabdogaJbqabaGccqqGGaaicqWGfbqrdaWgaaWcbaGaemiBaWMaemyyaeMaem4yamgabeaakmaalaaabaGaemitaWKaemyyaeMaem4yamgabaGaem4saS0aaSbaaSqaaiabdYgaSjabdggaHjabdogaJbqabaGccqGHRaWkcqWGmbatcqWGHbqycqWGJbWycqGGOaakcqaIXaqmcqGHRaWkdaWcaaqaaiabdIfayjabicdaWiabgkHiTiabdIfayjabdcfaqbqaaiabdUealnaaBaaaleaacqWGjbqscqWGfbqrcqWGjbqscqWGjbqscqWGbbqqaeqaaOGaemiwaGLaeGimaadaaiabcMcaPaaacqWGcbGqaaa@681D@

E˙Lac=rsynlac−(μ+kd) Elac=k3 gIlacgT−(μ+kd)ELac=k3rlacrlac+K3gT−(μ+kd) ELac
 MathType@MTEF@5@5@+=feaafiart1ev1aaatCvAUfKttLearuWrP9MDH5MBPbIqV92AaeXatLxBI9gBaebbnrfifHhDYfgasaacH8akY=wiFfYdH8Gipec8Eeeu0xXdbba9frFj0=OqFfea0dXdd9vqai=hGuQ8kuc9pgc9s8qqaq=dirpe0xb9q8qiLsFr0=vr0=vr0dc8meaabaqaciaacaGaaeqabaqabeGadaaakeaafaqaaeGadaaabaGafmyrauKbaiaadaWgaaWcbaGaemitaWKaemyyaeMaem4yamgabeaaaOqaaiabg2da9aqaaiabdkhaYnaaDaaaleaacqWGZbWCcqWG5bqEcqWGUbGBaeaacqWGSbaBcqWGHbqycqWGJbWyaaGccqGHsislcqGGOaakiiGacqWF8oqBcqGHRaWkcqWGRbWAdaWgaaWcbaGaemizaqgabeaakiabcMcaPiabbccaGiabdweafnaaBaaaleaacqWGSbaBcqWGHbqycqWGJbWyaeqaaOGaeyypa0Jaem4AaS2aaSbaaSqaaiabiodaZaqabaGccqqGGaaicqWGNbWzdaqhaaWcbaGaemysaKeabaGaemiBaWMaemyyaeMaem4yamgaaOGaem4zaC2aaSbaaSqaaiabdsfaubqabaGccqGHsislcqGGOaakcqWF8oqBcqGHRaWkcqWGRbWAdaWgaaWcbaGaemizaqgabeaakiabcMcaPiabdweafnaaBaaaleaacqWGmbatcqWGHbqycqWGJbWyaeqaaaGcbaaabaGaeyypa0dabaGaem4AaS2aaSbaaSqaaiabiodaZaqabaGcdaWcaaqaaiabdkhaYnaaBaaaleaacqWGSbaBcqWGHbqycqWGJbWyaeqaaaGcbaGaemOCai3aaSbaaSqaaiabdYgaSjabdggaHjabdogaJbqabaGccqGHRaWkcqWGlbWsdaWgaaWcbaGaeG4mamdabeaaaaGccqWGNbWzdaWgaaWcbaGaemivaqfabeaakiabgkHiTiabcIcaOiab=X7aTjabgUcaRiabdUgaRnaaBaaaleaacqWGKbazaeqaaOGaeiykaKIaeeiiaaIaemyrau0aaSbaaSqaaiabdYeamjabdggaHjabdogaJbqabaaaaaaa@885F@

**Figure 12:**
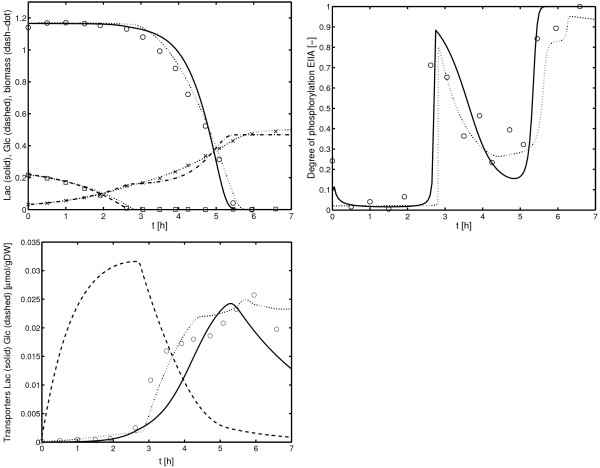
Glucose/lactose diauxic growth. Top: Time course of lactose, glucose and biomass. Middle: Time course of the degree of phosphorylation of EIIA^*Crr*^. Bottom: Time course of the activity of LacZ Dotted line are simulations with the original model [3] while solid lines are simulations with the new model.

As can be seen in the simulation in Figure 12, the time course of LacZ (right plot) can describe the experimental data, however, with less accuracy than the detailed model presented in [3].

### Comparison with a detailed model for catabolite repression

**Figure 13:**
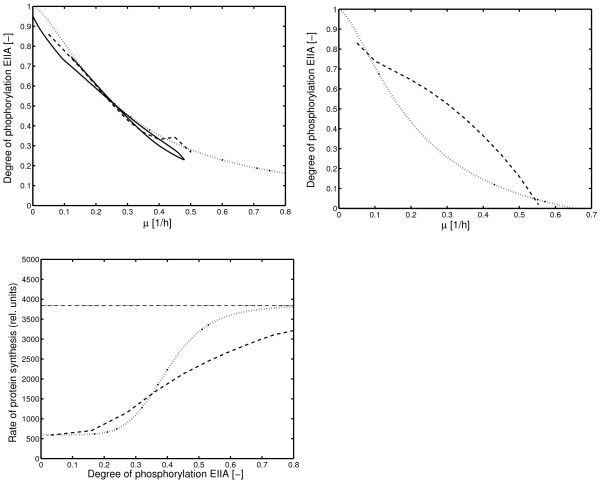
Comparison of simulation results of the proposed model with a more detailed model [3]. Top: Characteristic curve for non-PTS substrates. Solid line: Simulation of a batch experiment. (Figure 5 in the supplement in [3]). Values for the second growth phase, that is, growth on lactose are plotted. Dashed line: Simulation of a continuous fermentation with different values of the dilution rate with the model in [3]. Dotted line: Results with the proposed model. Middle: Characteristic curve for PTS substrates. Dashed line: Simulation of a continuous fermentation with different values of the dilution rate with the model in [3]. Dotted line: Results with the proposed model. Bottom: Characteristic curve to describe the relationship between the degree of phosphorylation of EIIA^*Crr *^and the rate of protein synthesis. Dashed line: Simulation of a continuous fermentation with different values of the dilution rate with the model in [3]. Dotted line: Results with the proposed model.

A very detailed model for catabolite repression was already introduced to describe a number of experiments under different conditions and with different strains [3]. However, especially for PTS uptake, only high growth rates were considered. Figure 13 compares the characteristic curve for the detailed model and the reduced model, introduced here and it can be seen, that, indeed, the detailed model fails to describe the experimental data for a broad range of the growth rate. The detailed model was also used to calculate a steady-state relationship for the transcription efficiency. As can be seen in the plot, again, for low growth rates, the detailed model fails to describe the experimental data.

## Conclusion

The paper presents evidence that a sensitive metabolic regulation at the PEP/pyruvate node results in a relationship between the phosphorylation state of EIIA^*Crr*^, an element of the sensory system PTS, and the specific growth rate *μ*. Under a variety of experimental conditions with a wild type strain and a mutant strain this relationship could be verified over a broad range of the growth rates, revealing the signaling and kinetic characteristics of the sensor. For the analysis of the system, a mathematical model with a small number of state variables (Table 1) was set up and based on an initial set of experimental data, model predictions were performed.

**Table 1:** Summary of the state variables of the model

State variable	Comment
B	biomass
Glc6P	extracellular glucose 6-phosphate
Glc	extracellular glucose
Lac	extracellular lactose
G6P	glucose 6-phosphate; represents the metabolites in the upper part of the glycolysis
PEP	phosphoenolpyruvate
Prv	pyruvate
XP	represents the phosphorylated form of the PTS proteins
	(EI, HPr, EIIA^*Crr*^)
E_*G*6*P*_	represents uptake system for glucose 6-phosphate (UhpT)
E_*Glc*_	represents uptake system for glucose (PtsG)
E_*Lac*_	represents uptake system for lactose (LacY)

Several kinetic properties determine the degree of phosphorylation of the PTS protein EIIA^*Crr*^. According to this study, the choice of the rate law for the pyruvate kinase is the most important one. While all other kinetic rate laws can be described with simple mass action rate laws, the pyruvate kinase has to be described with a power law kinetics. However, this choice is only true for a certain set of experimental conditions; considering only growth of the wild type on glucose, a simple rate law, as suggested by [19], is capable to describe experimental data. Based on a systems biology approach that considers different operational modi of the system and a directed stimulation of the system with respect to these modi, the present study shows that the simple rate law is not longer able to describe all experimental data. The core model comprises four reactions for glycolysis, pyruvate kinase, PTS, and drain to monomers. Parameters were determined by fitting experimental data from a wild type strain and a PtsG mutant strain. To deconstruct the results, a robustness analysis was performed that ranks the parameters according to the influence on the degree of phosphorylation of EIIA^*Crr *^in dependence on the growth rate. As expected, the biggest influence for both operational modi shows parameter *n*, that represents the influence of the feed-forward control of glucose 6-phosphate on pyruvate kinase. Furthermore, the overall concentration *X*_0 _of enzyme EIIA^*Crr *^has a big influence while the concentration of the other enzymes represented by *k*_*gly*_, *k*_*pyk*_, and *k*_*pdh *_is moderate and comparable to the influence of the remaining kinetic parameters *k*_*pts *_and *K*_*pts*_.

The feed-forward loop is a special motive (a regulatory pattern that is more present than others) described in detail for genetic systems [20]. Here, we found that this motive is essential for the transformation of a high incoming flux (high growth rate) into a low PEP/pyruvate ratio. To verify this, the internal metabolites PEP and pyruvate are measured. Since the errors for the procedure of the PEP and pyruvate measurement are rather high [4], the data shown in Figure 6 should be interpreted rather as a trend and not as quantitative measurements. Although measurements for small growth rates are not available, the PEP/pyruvate ratio could be predicted very well for growth rates in the range between 0.15 1/h and 0.7 1/h.

In engineering science, sensor or measurement systems are designed in such a way that they don't influence the system that is measured. This is called "free of retroactivity". Considering the PTS operational mode in comparison to the non-PTS mode the difference of the curves is due to the transport activity of the PTS. Hence, the sensor PTS is not free of retroactivity; however, for small growth rates, indicating a severe stress situation, the difference between the PTS mode and the non-PTS mode is negligible.

As representative of gluconeogenetic substrates, growth on acetate was considered. The fluxes are adjusted in such a way that a flux distribution published previously, is matched. Measurements of the degree of phosphorylation of EIIA^*Crr *^are in good agreement with the predicted values. The results also confirm that the Pps enzyme has only marginal influence on growth on acetate as described by [12]. However, the observation that a Pps mutant strain that grows simultaneously on glucose and acetate shows an extended lag phase could not be explained with model set up in this study.

The transcription efficiency according to Equation (37) revealed that the Hill-coefficient *n *= 6 is rather high. This might be due to several reasons: although the signal transduction pathway starting from EI and ending with Crp is rather short, several components and processes are involved. First cAMP is generated by the adenylate cyclase (Cya); second cAMP interacts with Crp to activate the transcription factor. Furthermore, transcription of Cya is also under control of Crp leading to a feedback loop. Since the kinetics of the individual steps are not yet characterized, the rather high Hill-coefficient can be seen as an overall measure of the sensitivity of the system. The kinetics determined are used to simulate the two dynamical experiments and a good agreement between the simulation data and the experimental data could be observed. This shows that not only the steady-state behavior can be reproduced well but also the dynamics of the sensor/actuator system.

The simplified scheme is used to analyze the growth behavior and the dynamics of *Escherichia coli *during growth on glucose/glucose 6-phosphate and on glucose/lactose. The model has to be extended to describe the kinetics of the transporters and the kinetics of gene expression for the relevant transporters. Since experimental data that characterize the *K*_*Glc *_value for glucose can be found in the literature, the respective value *K*_*EIIA *_for the degree of phosphorylation was determined by a simulation experiment with a random bi-bi double substrate kinetics, Equation (38), and experimental data from [4]. Parameters *k*_*max *_and *K*_*EIIA *_are determined by a least-square fit.

Growth on glucose/glucose 6-phosphate reveals the interesting observation that the concentration of the glucose transporter decreased during growth on glucose 6-phosphate. To match the experimental data, an inhibitory effect of the glucose 6-phosphate transporter UhpT on the glucose transporter PtsG was assumed and described with a simple kinetics. Previous studies revealed that the *ptsG *mRNA is under control by SgrS, a small RNA. It was shown that high levels of intracellular glucose 6-phosphate or fructose 6-phosphate lead to *ptsG *mRNA degradation [6,7]. Here, the model can be used to calculate the intracellular levels of glucose 6-phosphate and PEP in model variants with and without control of PtsG. As shown in Figure 11, no difference could be detected, indicating that the interaction between the two transporters is based on the activity of the glucose 6-phosphate transporter as suggested in [17]. Note, that to describe the time course of PtsG in Figure 10, three factors, namely the inhibition of PtsG by UhpT, induction of *ptsG *and global control of PtsG synthesis by Crp were taken into account and have to be adjusted very precisely.

A comparison with a detailed model for catabolite repression justifies the set up of the new model. Altough validated under different experimental conditons, the detailed model fails to describe growth on PTS carbohydrates on a broad range of the growth rate.

The approach is based on the development of a model with a minimal number of parameters that are necessary to describe the observations. Although some of the parameters have no defined mechanistic interpretation such models will facilitate the procedure of parameter analysis and estimation. The model is capable to simulate a broad range of experimental conditions and is suited for further studies on control systems on *E. coli *since it can be easily extended to describe other regulatory systems.

## Methods

For simulation of the algebraic system, solving the o.d.e. system, and parameter estimation MATLAB was used. Files to simulate the system with MATLAB and the experimental data can be found on a website [21]. For the experimental data, see the [Additional file 1] and a further manuscript from our group [4].

## Competing interests

The author(s) declares that there are no competing interests.

## Authors' contributions

AK performs modeling, model analysis and parameter estimation. KB performs the experiments. EDG conceived of the study, and participated in its design and coordination. All authors read and approved the manuscript.

## Supplementary Material

Additional file 1 Supplementary Information. The Supplementary Information includes the description of the experimental data and the values for the kinetic parameters of the mathematical model. Click here for file
