# Supplementary material for: Analysis of global control of Escherichia coli carbohydrate uptake
Source: BMC Syst Biol. 2007 Sep 13;1:42. doi: 10.1186/1752-0509-1-42 (PMC2148058; doi:10.1186/1752-0509-1-42)
Supplement: Additional file 1 — Supplementary Information. The Supplementary Information includes the description of the experimental data and the values for the kinetic parameters of the mathematical model. [file 1752-0509-1-42-S1.pdf]

Analysis of global control of *Escherichia coli* carbohydrate uptake – Supporting Information

A. Kremling, K. Bettenbrock and E.D. Gilles

Systems Biology Group, Max-Planck-Institut für Dynamik komplexer technischer Systeme, Magdeburg, Germany, Email: kremling@mpi-magdeburg.mpg.de

Material, experimental data and parameter estimation

Strains

All strains used were isogenic derivatives of LJ110 (wild type), constructed by standard P1 *kc* transduction techniques (Kremling et al., 2001). Strains and the relevant mutations are listed in Table 1.

Table 1: Strains.

**Strain**	**Genotype**	**Origin**
LJ110	W3110, F^-, Fnr^+	(Zeppenfeld et al., 2000)
BKG47	LJ110 Δ(*p*tsG)::*cat*	(Kremling et al., 2001)
LZ110	LZ110 F'8 $ptsG_{o,p}\phi$ *lacZ*	(Zeppenfeld et al., 2000)

Experimental data

Experimental data are taken from Bettenbrock et al. (2006 2007) and are described in detail there. Using a wild type strain (LJ110) and various mutant strains the experiments are performed under various growth conditions and different growth substrates. To follow the dynamics of the phosphorylation of EIIA^{Crr} of the PTS during diauxic growth, the calculation of the growth rate based on the measured biomass concentration results in large fluctuations since a differentiation has to be done. Instead we used the mathematical model in (Bettenbrock et al., 2006) to simulate the specific growth rate μ. Table 2 collocate the data for the growth rate and the degree of phosphorylation of EIIA^{Crr}. Experimental data for different carbon sources are summarized in Table 3. Standard deviations for the data are given in Bettenbrock et al. (2007). Here, furthermore, the PEP/pyruvate ratio, and expression data are given. Gene expression was determined with a Crp independent gene (*scrK*) and a Crp dependent gene (*scrY*). Values are multiplied with the specific growth rate, to calculate the rate of protein synthesis.

Besides batch experiments, continuous culture were performed to follow the degree of phosphorylation of EIIA^{Crr} for different growth rates but for one carbon source (Table 4 and 5).

Table 2: Experimental data. Growth rate and degree of phosphorylation during diauxic growth (Bettenbrock et al., 2006)

**C source**	μ **[1/h]**	$\mathbf{EIIA}^P$ [-]	strain/source
Glc/Lac	0.54	0.02	
	0.54	0.04	
	0.54	0.00	
	0.55	0.07	
	0.17	0.65	
	0.31	0.36	
	0.39	0.46	
	0.43	0.23	
	0.45	0.39	
	0.44	0.32	
	0.13	0.89	
	0.001	1.00	LJ110, Figure 5, (Bettenbrock et al., 2006)
Glc/Lac	0.66	0.04	
	0.61	0.04	
	0.58	0.03	
	0.56	0.05	
	0.54	0.20	
	0.34	0.50	
	0.43	0.37	
	0.48	0.34	
	0.49	0.32	
	0.48	0.40	LJ110, Figure 6, (Bettenbrock et al., 2006)
Glc/Gly	0.30	0.66	
	0.31	0.44	
	0.31	0.39	
	0.27	0.40	LZ110, , Figure 12,(Bettenbrock et al., 2006)
Glc/Lac	0.55	0.31	
	0.52	0.28	
	0.30	0.72	
	0.31	0.76	
	0.31	0.77	
	0.00	0.97	BKG47 , Figure 14,(Bettenbrock et al., 2006)

Table 3: Experimental data Bettenbrock et al. (2007). Growth rate, degree of phosphorylation, PEP/pyruvate ratio, and expression data during exponential growth of different carbon sources diauxic growth. Data for gene expression was determined in an independent experiment. Therefore, the growth rate for these experiments are designed with μ^2.

**C source**	μ **[1/h]**	**EIIA**^{P} **[-]**	**PEP/prv [-]**	μ^2 **[1/h]**	**ex. [rel. units]**	
					ScrK	ScrY
D-glucose 6-phosphate	0.74	0.20	0.21	0.72	4233	82
D-glucose	0.68	0.05	0.15	0.62	3470	788
Sucrose	0.65	0.10	0.45	n.d.	n.d.	
Lactose	0.6	0.17	0.12	0.6	4286	584
N-acetyl-d-glucosamine	0.57	0.17	n.d.	0.57	3316	596
D-mannitol	0.58	0.14	0.22	0.59	3797	754
l-arabinose	0.51	0.25	n.d.	0.55	3672	1119
D-gluconate	0.50	0.30	0.03	0.54	3741	875
Maltose	0.48	0.41	0.27	0.5	3650	1668
sn-glycerol	0.43	0.47	n.d.	0.42	4629	3986
D-fructose	0.41	0.24	0.4	0.45	3981	3542
Succinate	0.35	0.65	1.07	0.38	5461	8131
acetate	0.17	0.33	1.3	0.13	1561	2600
D-mannose	0.15	0.48	1.4	0.18	4085	4713
D-glucosamine	0.12	0.64	n.d	0.21	4185	5029
D-galactose	0.28	0.59	n.d.	n.d.	n.d.	

Table 4: Experimental data. Residual glucose concentration and degree of phosphorylation during the start phase of a continuous culture. Data are given for three independent experiments. Reproducibility and protocols according to Bettenbrock et al. (2006)

**Glc [g/l]**	**EIIA^P [-]**	**Glc [g/l]**	**EIIA^P [-]**	**Glc [g/l]**	**EIIA^P [-]**
0.1190	0	0.1719	0.11	0.0939	0
0.1070	0.02	0.1652	0.10	0.0820	0
0.1120	0.03	0.1396	0.10	0.0790	0
0.1070	0.04	0.1140	0.10	0.0771	0
0.0814	0.05	0.1083	0.07	0.0718	0
0.0630	0.06	0.1016	0.09	0.0657	0
0.0410	0.07	0.0978	0.10	0.0563	0
0.0293	0.08	0.0883	0.11	0.0507	0
0.0105	0.09	0.0817	0.13	0.0479	0.14
0.0081	0.09	0.0674	0.10	0.0410	0.13
0.0039	0.23	0.0618	0.12	0.0324	0.10
0.0037	0.52	0.0543	0.07	0.0258	0.15
0.0031	0.53	0.0536	0.08	0.0192	0.18
0.0008	0.67	0.0424	0.08	0.0121	0.34
0.0004	0.71	0.0269	0.16	0.0077	0.31
N.D.	0.74	0.0266	0.08	0.0049	0.52
N.D.	0.93	0.0158	0.13	0.0039	0.35
0.0004	0.93	0.0083	0.15	0.0035	0.42
0.0002	0.92	0.0030	0.42	0.0032	0.33
		0.0004	0.54	0.0031	0.48
		0.0007	0.43	0.0026	0.49
		0.0014	0.34	0.0021	0.50
		0.0011	0.35	0.0018	0.54
		0.0009	0.29	0.0020	0.63
		0.0002	0.86	0.0011	0.58
		0.0001	0.87		

Table 5: Experimental data. Residual mannitol concentration and degree of phosphorylation during the start phase of a continuous culture. Reproducibility and protocols according to Bettenbrock et al. (2006).

**Mtl [g/l]**	**EIIA**^{P} [-]
0.0888	0.09
0.0748	0.09
0.0691	0.19
0.0632	0.10
0.0566	0.07
0.0427	0.11
0.0334	0.20
0.0274	0.15
0.0217	0.14
0.0148	0.09
0.0078	0.23
0.0035	0.87
0.0010	0.86
0.0001	0.89
0.0000	0.90
0.0000	0.90
0.0000	0.84
0.0000	0.90
0.0000	0.96
0.0000	0.90
0.0000	0.94
0.0000	0.95
0.0000	0.92
0.0000	0.92
0.0001	0.92
0.0001	0.91
0.0001	0.90
0.0001	0.92
0.0001	0.91
0.0001	0.86

Parameters - model uncertainties and parameter uncertainties

Model parameters are summarized in Table 6. Although the model is minimal model from

Table 6: Parameter values. Literature [1](Bettenbrock et al., 2006)

	**value**	**source**
k_{gly}	$2.80 \cdot 10^4$ [1/h]	estimated
k_{pdh}	$5.50 \cdot 10^3$ [1/h]	estimated
k_{pts}	$1.86 \cdot 10^5$ [1/(μmol/gDW) h]	estimated
K_{pts}	0.7 [-]	literature[1]
k_{pyk}	$9.39 \cdot 10^5$ [1/(μmol/gDW)^3 h]	estimated
n	2 [-]	empirical
m	1 [-]	empirical
k_{pps}	$0.5 \cdot 10^2$ [μmol/gDW h]	adjusted
h_1	0.1 [-]	adjusted
h_2	0.07 [-]	adjusted
k_{glu}	$2.80 \cdot 10^4$ [1/h]	assumed equal k_{gly}
k_{bio}	$5.0 \cdot 10^3$ [1/h]	adjusted
$k_{pts \cdot up}$	$2.70 \cdot 10^8$ [1/h]	estimated
K_{EIIAP}	12 [μmol/gDW]	estimated
K_{glc}	0.12 [g/l]	literature[1]
k_{syn}	$3.26 \cdot 10^3$ [-]	estimated
k_b	600 [-]	estimated
K	0.4 [μmol/gDW]	estimated
k_{g6p}	$2.80 \cdot 10^6$ [1/h]	estimated
K_{g6p}	0.1 [g/l]	estimated
X_0	0.1 [μmol/gDW]	literature[1]

the biological point of view, there are different parameter combinations that lead to nearly equal values of the objective function.

Table 6 cont.

	**value**	**source**
k_{lac}	$5.40 \cdot 10^5$ [1/h]	estimated
K_{lac}	0.13 [g/l]	estimated
k_d	0.4 [1/h]	estimated
K_{IEIIA}	5.0 [-]	estimated
K_I	$1.25 \cdot 10^{-4}$ [μmol/gDW]	estimated
k_1	$1.0 \cdot 10^{-5}$ [μmol/gDW h]	adjusted
K_1	$3.0 \cdot 10^3$ [μmol/gDW h]	adjusted
k_2	$1.0 \cdot 10^{-4}$ [μmol/gDW h]	adjusted
K_2	$2.8 \cdot 10^3$ [μmol/gDW h]	adjusted
k_3	$1.6 \cdot 10^{-4}$ [μmol/gDW h]	adjusted
K_3	$1.5 \cdot 10^4$ [μmol/gDW h]	adjusted
Y_{lac}	$0.924 \cdot 10^{-4}$ [gDW/μmol]	experimental
Y_{g6p}	$0.625 \cdot 10^{-4}$ [gDW/μmol]	experimental
Y_{glc}	$1.0 \cdot 10^{-4}$ [gDW/μmol]	experimental

The estimated parameter represent therefore a local minima of the objective function. (MATLAB was used to estimate the parameters, different solvers were used and compared). Therefore, and based on the fact that (i) only values for high growth rates for growth on glucose were available, Figure 3 and (ii) parameter k_{pts} has no sensitivity in the non PTS case, Figure 4, the estimation of the parameter uncertainties is not a trivial task. Here, we used a statistical method, the bootstrapping Joshi et al. (2006), to determine parameter uncertainties. The method surmounts the theoretical limitations (e.g. the Fisher-Information matrix gives only a lower bound for the parameter variances in case of systems that are nonlinear in parameters) by assessing the uncertainties in statistics with data from finite samples. Like a Monte-Carlo method, the bootstrap uses stochastic elements and repeated simulations to analyze the properties of the system under consideration.

Briefly, the analysis is performed in such a way, that an initial set of experimental data $\mathbf{S}$ is used as a data base. Performing parameter estimation result in a first set of parameters to assess the model quality. Due to measurement errors the repetition of the experiment leads to a slightly different set of data $\mathbf{S_1}$ and therefore to a different set of estimated parameters. The bootstrap approach now uses a large set of B-times replicated experimental

data $\mathbf{S_1}, \mathbf{S_2}, \mathbf{S_3} \cdots \mathbf{S_B}$ to calculate statistical properties of the resulting distribution of the (re)-estimated set of parameters and σ values. Table 7 summarizes the results for the 4 parameters.

Table 7: Parameter confidence regions.

	**estimated value**	**confidence region**
k_{gly}	$2.80{\cdot}10^4$ [1/h]	2.79 – 2.98 $\cdot 10^4$
k_{pdh}	$5.50{\cdot}10^3$ [1/h]	5.35 – 6.70 $\cdot 10^3$
k_{pts}	$1.86{\cdot}10^5$ [1/(μmol/gDW) h]	1.22 – 3.26 $\cdot 10^5$
k_{pyk}	$9.39{\cdot}10^5$ [1/(μmol/gDW)^3 h]	9.25 – $9.50{\cdot}10^5$

The confidence region for the simulation is based on the linearization around the estimated parameters. The calculation of the Fisher-Information-Matrix W_{ex} taking into account only the measured data point results in a matrix with rank equal 3. Therefore, the analysis has to be restricted to three parameters (X_0,K_{pts}, n) in matrix W_{ex}. The confidence region for a value of $\hat{XP}_j$ is determined with

$$\hat{XP}_j = XP_j \ \pm \ 2 \ \sqrt{\hat{\sigma}_j^2 \ \left(1 + \underline{w}_j \ W_{ex}^{-1} \ \underline{w}_j^T\right)} \qquad (1)$$

with $\underline{w}_j$ is the vector of the regressor with elements $w_i = \frac{dXP}{dp_i}$ for the linearized model for fixed values μ_j of the growth rate.

References

K. Bettenbrock, S. Fischer, A. Kremling, K. Jahreis, T. Sauter, and E. D. Gilles. A quantitative approach to catabolite repression in *Escherichia coli*. *J. Biol.Chem.*, 281:2578–2584, 2006.

K. Bettenbrock, T. Sauter, K. Jahreis, A. Kremling, J. W. Lengeler, and E. D. Gilles. Analysis of the correlation between growth rate, EIIA^{Crr} (EIIA^{Glc}) phosphorylation levels and intracellular cAMP levels in *Escherichia coli* K-12. *J. Bacteriology*, 2007. Accepted.

M. Joshi, A. Seidel-Morgenstern, and A. Kremling. Exploiting the bootstrap method for quantifying parameter confidence intervals in dynamical systems. *Metab. Eng.*, 8(5):447–455, 2006.

A. Kremling, K. Bettenbrock, B. Laube, K. Jahreis, J.W. Lengeler, and E.D. Gilles. The organization of metabolic reaction networks: III. Application for diauxic growth on glucose and lactose. *Metab. Eng.*, 3(4):362–379, 2001.

T. Zeppenfeld, C. Larisch, J.W. Lengeler, and K. Jahreis. Glucose transporter mutants of *Escherichia coli* K-12 with changes in substrate recognition of the IICB^{Glc} and induction behavior of the *ptsG* gene. *J. Bacteriol.*, 182:4443–4452, 2000.
